# *Plasmodium falciparum* gametocyte production correlates with genetic markers of parasite replication but is not influenced by experimental exposure to mosquito biting

**DOI:** 10.1016/j.ebiom.2024.105190

**Published:** 2024-06-19

**Authors:** Sara Lynn Blanken, Aissata Barry, Kjerstin Lanke, Moussa Guelbeogo, Alphonse Ouedraogo, Issiaka Soulama, Sam Aboubacar Coulibaly, Karina Teelen, Wouter Graumans, Elin Dumont, Will Stone, Jordache Ramjith, Matthias Marti, Carolina M. Andrade, Chris Drakeley, Katharine Collins, Alfred Tiono, Teun Bousema

**Affiliations:** a Department of Medical Microbiology, Radboud University Medical Centre, Nijmegen, the Netherlands; b Groupe de Recherche Action en Santé (GRAS), Ouagadougou, Burkina Faso; c Centre National de Recherche et de Formation sur le Paludisme (CNRFP), Ouagadougou 01, Burkina Faso; d Department of Immunology and Infection, London School of Hygiene and Tropical Medicine, London, UK; e Wellcome Centre for Integrative Parasitology, Institute of Infection and Immunity, University of Glasgow, Glasgow, Scotland, UK; f MRC International Statistics and Epidemiology Group, London School of Hygiene and Tropical Medicine, London, UK

**Keywords:** Malaria transmission, *Plasmodium falciparum*, Gametocyte commitment, Genetic markers, Mosquito exposure, *Anopheles*

## Abstract

**Background:**

*Plasmodium* blood-stage parasites balance asexual multiplication with gametocyte development. Few studies link these dynamics with parasite genetic markers in vivo; even fewer in longitudinally monitored infections. Environmental influences on gametocyte formation, such as mosquito exposure, may influence the parasite's investment in gametocyte production.

**Methods:**

We investigated gametocyte production and asexual multiplication in two *Plasmodium falciparum* infected populations; a controlled human malaria infection (CHMI) study and a 28-day observational study in naturally infected individuals in Burkina Faso with controlled mosquito exposure. We measured gene transcript levels previously related to gametocyte formation (*ap2-g, surfin1.2, surfin13.1, gexp-2*) or inhibition of asexual multiplication (*sir2a*) and compared transcript levels to ring-stage parasite and mature gametocyte densities.

**Findings:**

Three of the five markers (*ap2-g, surfin1.2, surfin13.1*) predicted peak gametocytaemia in the CHMI study. An increase in all five markers in natural infections was associated with an increase in mature gametocytes 14 days later; the effect of *sir2a* on future gametocytes was strongest (fold change = 1.65, IQR = 1.22–2.24, P = 0.004). Mosquito exposure was not associated with markers of gametocyte formation (*ap2-g* P = 0.277; *sir2a* P = 0.499) or carriage of mature gametocytes (P = 0.379).

**Interpretation:**

All five parasite genetic markers predicted gametocyte formation over a single cycle of gametocyte formation and maturation in vivo; *sir2a* and *ap2-g* were most closely associated with gametocyte growth dynamics. We observed no evidence to support the hypothesis that exposure to *Anopheles* mosquito bites stimulates gametocyte formation.

**Funding:**

This work was funded by the 10.13039/100000865Bill & Melinda Gates Foundation (INDIE OPP1173572), the European Research Council fellowship (ERC-CoG 864180) and UKRI Medical Research Council (MR/T016272/1) and Wellcome Center (218676/Z/19/Z).

**Research in context**

**Evidence before this study**

Field studies attempting to quantify gametocyte commitment are challenged by the sequestration of committed parasites away from the circulation. Genetic markers of gametocyte commitment or early gametocyte formation may predict the formation of gametocytes and their subsequent release into the blood circulation from the bone marrow and spleen. We performed a PubMed search without language restrictions for studies published up to September 2023 on gametocyte commitment in relation to parasite gene transcription. We used the following search terms (malaria) AND (falciparum) AND ((differentiation) OR (commitment) OR (development) OR (gametocytogenesis) OR (conversion)) AND ((gametocyte) OR (sexual)) AND ((gene) OR (transcription)).

Several in vitro transcriptomic studies report genes associated with sexual differentiation in *Plasmodium falciparum*. By creating a genetic mutant parasite in 3D7 parasites, a study in 2014 observed the transcription factor *ap2-g* to be essential for sexual differentiation. Several other in vitro studies confirmed the importance of *ap2-g* whilst also identifying new candidates including *gexp-5, gexp-2, msrp1, pfpeg4*, *pfg27, gdv1* and antisense RNA *gdv1*. Additional studies used ex vivo gametocyte production assays to find genes upregulated in samples with high gametocyte conversion rates. These studies identified new candidate markers including *surfin1.2* and *surfin13.1*.

Few studies have tested the performance of genetic markers of gametocyte production in vivo and data from longitudinally monitored natural infections is even more scarce. We found two cross-sectional studies assessing genetic transcripts in vivo; one study in Kenyan adults observed that *sir2a*, an inhibitor of DNA multiplication, was associated with higher concurrent gametocytaemia. One other study indirectly studied gametocyte formation in relation to genetic markers and identified increased *sir2a* transcripts in afebrile infections from Mozambique and reported similar gametocyte transcripts in age- and parasite density matched febrile infections. We found three studies that used a limited number of genetic markers to assess gametocyte formation in a longitudinal setting: one controlled human malaria infection study in malaria naïve adults from the Netherlands associated increased transcripts of *ap2-g* with peak gametocytaemia after treatment. One study on patients with malaria from Mozambique, Burkina Faso and Vietnam reported a poor correlation between *ap2-g* expression and gametocyte carriage one to two weeks after malaria treatment. We found only one longitudinal study in naturally acquired untreated malaria infections that tested the performance of gametocyte commitment or early gametocyte markers *ap2-g* and *gexp-5* and observed a positive correlation with gametocyte densities 14 days later.

The literature search described here was used for the selection of markers assessed in the present study. Selected genetic markers were used to investigate the long-standing hypothesis that *Anopheles* exposure could stimulate gametocyte commitment in the field. This hypothesis finds its origin in observations from non-human malaria models and epidemiological correlations in areas of seasonal malaria transmission, yet no studies were identified that tested this hypothesis in longitudinally monitored infections or with molecular markers of gametocyte formation.

**Added value of this study**

This study combines data from a controlled human malaria infection (CHMI) study in the Netherlands and a longitudinal observational study in Burkina Faso. The CHMI study involved 24 healthy adults who were infected and subsequently treated to clear asexual parasites, whilst allowing gametocytes to develop. This study was complemented with a longitudinal study in Burkina Faso that was specifically designed to examine gametocyte commitment markers in relation to gametocyte formation. The field study was conducted in the dry season when incident malaria infections are unlikely; 120 individuals with chronic asymptomatic *Plasmodium falciparum* infections were recruited and followed up at four visits spanning 28 days. Half-way through their follow-up, 59 individuals (59/120, 49.2%) were exposed to 60 uninfected *Anopheles* mosquito bites as part of an assessment of their infectivity to mosquitoes; subsequent gametocyte formation was compared to non-exposed parasite carriers. Both studies were used to assess the relationship between genetic marker expression and parasite dynamics, while the study in Burkina Faso also enabled us to formally test the hypothesis that *Anopheles* mosquito exposure may stimulate gametocyte commitment.

Our findings demonstrate three markers to be associated with peak gametocytaemia in the CHMI study and five markers to be predictive of in vivo gametocyte formation in the Burkina Faso study. Transcripts of *ap2-g* and *gexp-2* were positively associated with gametocyte densities 14 days later. Compared to unexposed controls, individuals who were exposed to *Anopheles* bites did not show an increase in gametocyte commitment marker expression or formation of mature gametocytes. Data were too sparse to allow for a statement on the impact of mosquito exposure on mosquito infection rates.

**Implications of all the available evidence**

Our study adds to the existing transcriptomic data on *Plasmodium falciparum* gametocyte commitment, that identified candidate genes associated with gametocyte commitment, by directly assessing the comparative performance of gene transcripts as markers of gametocyte formation in two distinct cohorts with longitudinally monitored infections. Our findings further add to the scarce evidence on the effect of mosquito exposure on *Plasmodium falciparum* gametocyte formation. We report that a single high mosquito exposure event does not result in increased gametocyte commitment in chronic, naturally acquired infections.

## Introduction

Malaria remains a major global health problem and comprehensive approaches to accurately define the malaria infectious reservoir may assist in effectively disrupting transmission.^1^, ^2^, ^3^ Human to mosquito *Plasmodium* transmission is mediated by non-replicative sexual stages of the parasite called gametocytes. Gametocytes are formed during a blood-stage infection when a fraction of asexual parasites divert from asexual multiplication and commit to gametocyte formation instead.^4^ Committed parasites are sequestered to the extravascular tissues of the bone marrow, where they develop through five stages (I to V) of gametocyte maturation. After 9–12 days, mature stage V female and male gametocytes are released into the human blood circulation.^5^ Mature gametocytes can subsequently be taken up by a blood-feeding *Anopheles* mosquito and can render the mosquito infectious upon its next bloodmeal.

Whilst there appears to be low-level stochastic production of gametocytes in vitro,^6^ gametocyte production varies considerably between^7^^,^^8^ and during^9^ different natural infections. Factors that may influence gametocyte production include host hematological factors,^10^ infection duration,^7^^,^^8^ and human immune responses.^11^, ^12^, ^13^ Environmental cues could also influence parasite transmission, as demonstrated for other vector-borne diseases. The cyclical rise and fall in microfilaremia such as *Wuchereria bancrofti* is associated with the presence of *Culicidae* vectors,^14^ and seasonal changes in microfilaremia may be linked with vector abundance.^15^ Seasonal changes in the availability of *Anopheles* mosquitoes could also influence gametocyte production.^16^ Exposure to mosquito bites has been associated with gametocyte production for the rodent malaria parasite species *Plasmodium chabaudi*,^17^ although evidence is equivocal.^18^ Birds infected with avian malaria infected more mosquitoes when they had previously been experimentally exposed to uninfected mosquito bites.^19^ *Anopheles* saliva induces quantifiable immune responses in humans, which can be specific to genus and species.^20^^,^^21^ Whilst unproven, mosquito saliva-induced gametocytogenesis could explain the observed peak in gametocyte prevalence at the beginning of the wet season^22^^,^^23^ and form an attractive strategy for parasites that survived the long dry season to allow onward transmission and sexual reproduction once vector numbers rise.

Attempts to quantify gametocyte commitment have mainly focused on the molecular detection of genetic commitment markers. Ex vivo gametocyte production assays that compared transcriptomes of patients with malaria with either high- or low-gametocyte-conversion rates^4^^,^^24^ identified genes upregulated in high-gametocyte-conversion samples, among which were the transcription factor *ap2-g*, previously associated with sexual commitment,^6^^,^^25^ and new candidates *surfin1.2* and *surfin13.1*.^24^ Additional in vitro studies showed that several other genetic markers were associated with gametocyte commitment or early development, including *gdv1*,^26^, ^27^, ^28^ *gexp-5*,^24^^,^^29^, ^30^, ^31^ *gexp-2*,^32^, ^33^, ^34^, ^35^ *msrp1*,^4^^,^^24^^,^^36^ *pfpeg4*,^31^^,^^37^^,^^38^ *pfg27*.^38^, ^39^, ^40^ *Plasmodium* parasites must balance gametocyte formation with asexual multiplication to ensure transmission to mosquitoes and survival in the host. Inhibitors of DNA multiplication and asexual proliferation, such as *sir2a*,^41^ have also been associated with increased gametocyte formation in vitro^42^; a recent cross-sectional study in Kenya also reported a positive correlation between *sir2a* and *ap2-g* transcript levels.^13^ Few studies report findings on the association between gene expression and asexual/sexual stage parasite dynamics in vivo, with an even more pronounced scarcity of evidence derived from longitudinal follow-up studies.^7^ Longitudinal studies are required to formally confirm and quantify the predictive value of marker transcripts for gametocyte formation potential.

Here, we investigated the relationship between transcript levels of gametocyte formation associated genes and in vivo parasite dynamics in two malaria infected cohorts, including a longitudinal study in Burkina Faso with natural infections and a controlled human malaria infection (CHMI) study in the Netherlands. We also explored whether a single high exposure to uninfected *Anopheles* mosquito bites stimulates gametocyte commitment and the formation of mature gametocytes.

## Methods

### Study populations and procedures

Samples from two malaria-infected populations were used. The first study was a previously published CHMI study conducted between May and November 2018 at Radboud University Medical Center in Nijmegen, the Netherlands.^43^^,^^44^ Briefly, 24 participants who were malaria-naïve (15 female and 9 male sex assigned at birth) were enrolled in two groups of equal sizes (n = 12) and infected by bites from five *P. falciparum* 3D7-infected mosquitoes (MB inoculation with culture started from 3D7 cell bank described in^45^) or by intravenous injection with ∼2800 *P. falciparum* 3D7-infected erythrocytes (IBS inoculation). To allow gametocyte formation but attenuate asexual infections, participants were treated with a single low dose (480 mg) of piperaquine treatment on day 8 after inoculation (IBS inoculation) or when parasite density reached 5000/mL (MB inoculation). Samples were collected at the moment of treatment and related to the peak gametocyte density observed during follow-up.

The second study took place between March and July 2021 in Saponé, 45 km Southwest of Ouagadougou, Burkina Faso. This is a region of intense and highly seasonal malaria transmission from June to October, with 71% of the population infected with *P. falciparum* at the end of the transmission season (November) and 25% still infected towards the end of the dry season (April).^46^ Individuals aged 5 years or above were eligible for screening, which consisted of a rapid diagnostic test (RDT) and a blood smear if the RDT was positive. Asymptomatic individuals with a minimum parasite density of 100 parasites/μL by microscopy were recruited into a longitudinal study where each participant was invited to the clinic on days 0 (enrolment), 14, 20 and 28 for clinical examination and venipuncture. Baseline concentrations of Human C-reactive protein (CRP) were measured in diluted plasma samples (1/2000) using an enzyme-linked immunosorbent assay (ELISA) with anti-CRP (DY1707; DuoSet).^47^ CRP concentration was calculated in mg/L from the standard curve; a CRP level of 10 mg/L was used as a cut-off for an acute phase response.^48^ Acquired immunity in response to previous *P. falciparum* exposure was assessed at baseline using a panel of blood-stage malaria antigens on a Luminex platform.^49^ This panel included antigens related to cumulative exposure (apical membrane antigen 1 (AMA-1), glutamate-rich protein (GLURP.R2), merozoite surface protein 1.19 (MSP-1.19)), and antigens related to exposure in the past six months (reticulocyte-binding protein homologue (Rh2.2030), early transcribed membrane protein (Etramp5.Ag1), and gametocyte exported protein (GEXP18)).

On day 14 and day 28, heparinized venous blood of all participants from Burkina Faso was offered to locally reared *Anopheles gambiae* mosquitoes to determine transmission potential using established membrane feeding methods.^7^ On day 14, 49.2% of the individuals (n = 59) also participated in direct skin feeding assays as a more sensitive method for transmission potential.^50^ For this assay, individuals were exposed for 25 min to two cups of 30 uninfected *Anopheles gambiae* mosquitoes (‘mosquito exposed cohort’), the remaining individuals did not participate in skin feeding (‘control cohort’) at this time-point. On day 28, all participants participated in membrane feeding. Natural mosquito exposure, assumed to be very low since the study was conducted in the dry season, was quantified by pyrethrum spray sheet collections in participant's households on day 0 and day 14 of the study period. Mosquito exposure was further explored by ELISA, quantifying human IgG antibodies against salivary gland protein gSG6^20^ at day 14 (baseline of mosquito exposure) and at day 28 (14 days after mosquito exposure).

### Ethics

The Nijmegen CHMI trial protocol was approved by the central committee for research involving human subjects and the Western Institutional Review Board, and it was registered at ClinicalTrails.gov (identifier NCT03454048) and EudraCT (identifier 2017-00040005-40). All participants provided written informed consent. The ethics committee of the London School of Health and Tropical Medicine (#22638) and the Burkina Faso national medical ethics committees provided ethical approval (#2020-9-187) for the longitudinal follow-up study in Burkina Faso. Written informed consent by the individual or a legal guardian was obtained from all eligible study participants before screening and again before official enrolment.

### Parasite quantification

Ring-stage parasites and gametocytes were quantified on the day of treatment of CHMI participants and on four time-points of the Burkina Faso cohort (day 0, 14, 20, and 28). For CHMI participants, gametocyte and parasite density was also determined on daily visits from day 4 until day 29 after treatment. Using automated extraction, total nucleic acids were extracted from 100 μL whole blood for the Burkina Faso cohort and 250 μL for the CHMI cohort both stored in RNA preservative (RNAprotect Cell Reagent; Qiagen). For CHMI participants, parasitaemia was quantified using 18S quantitative polymerase chain reaction (qPCR) as described previously.^43^ For both cohorts, ring-stage parasites were quantified using the *sbp-1* marker in a quantitative real time reverse transcriptase polymerase chain reaction (qRT-PCR) using the GoTaq qPCR Master Mix (Promega).^30^ For the Burkina Faso cohort, marker transcripts of *uce* (ubiquiting conjugating enzyme) were quantified from DNAse I RQ (Promega) treated material using the GoTaq qPCR Master Mix (Promega) as an alternative housekeeping gene.^51^ Cycling conditions for GoTaq mix were activation at 95C for 2 min, followed by 40 cycles at 95C for 15 s and at 60C for 1 min. Mature gametocyte density was assessed by qRT-PCR targeting female (*CCp4*) and male (*PfMGET*) mRNA transcripts using Luna One Step mix (New England Biolabs, USA), with a lower limit of detection of 0.01 gametocytes/μL.^52^ For Luna One Step mix, cycling conditions were 15 min at 55C, followed by 1 min at 95C and 45 cycles at 95C for 10 s and at 60C for 1 min. Total gametocyte density was adjusted for background transcripts in asexual parasites.^52^ Primer sequences and concentrations are available in Supplementary Table S1.Primer efficiency was determined using serial dilutions of a linear plasmid and ranged from 91.1% to 109% (Supplemental Data).

Detection of the single copy polymorphic *msp2* gene was used to determine multiplicity of infection in the Burkina Faso cohort. Using extracted nucleic acids from one time-point (day 0 or 14) per cohort participant, the entire *msp2* gene was first amplified in a nested PCR, followed by amplification of the allele strain-specific gene variant type FC27 or 3D7.^53^ Products were run on an agarose gel to detect samples positive and negative for the FC27 or 3D7 families. Infections were classified as polyclonal if i) multiple bands for one family were detected, or ii) one or more bands were detected for both families.

### Detection of marker transcripts for asexual multiplication, gametocyte commitment and development

Extracted nucleic acids (described above) were used directly for cDNA preparation for detection of transcripts from two intron-spanning genes (*gdv1, gexp-5*) or first treated with DNase I RQ1 (Promega) for 9 non-intron spanning genes (*ap2-g*, *sir2a, surfin1.2, surfin13.1*, *gexp-2, pfpeg4, msrp1, gdv1,* asRNA *gdv1*). Using the High Capacity cDNA Reverse Transcription Kit (Applied Biosystems), cDNA was prepared and combined with GoTaq qPCR Master Mix (Promega). Cycling conditions were as described above for the GoTaq qPCR Master Mix. Transcript levels were quantified as transcripts per μL against a plasmid standard-curve. The ratio of marker transcript to *sbp-1* was used as an indication of the proportion of sexually committed ring-stage parasites. Primer sequences and concentrations of the 11 markers are available in Supplementary Table S2. For the five markers selected for further analysis, primer efficiency was tested using serial dilution of a linear plasmid and ranged from 102% to 107% (Supplemental Data).

### In vitro parasite culture and genetic marker selection

*Plasmodium falciparum* NF54 strain (clone 3D7)^54^ was cultured in a semi-automated system as described previously.^55^ Briefly, we obtained asexual blood-stage parasites and gametocytes through synchronous culture in vitro. Blood-stage parasites were harvested at 10, 20, 30, and 40 h post Percoll synchronization to collect early and late-ring stage parasites, mature trophozoites, and schizonts, respectively. NF54 culture material was treated with N-acetylglucosamine (NAG) to eliminate asexual parasites and obtain mature gametocytes at day 16 post synchronization. With this material, we tested performance of 11 previously reported genetic markers using qRT-PCR as described above (Supplementary Table S3).^6^^,^^24^^,^^26^^,^^29^^,^^34^^,^^38^^,^^42^^,^^56^ Transcript levels of five down-selected markers were determined on the day of treatment of CHMI participants and on days 0, 14, and 20 of the Burkina Faso cohort by qRT-PCR. Samples with extraction failures (n = 6) and reactions with incorrect melt-curves were excluded from analyses.

### Statistical analyses

The nonparametric Mann–Whitney U test was used to compare continuous variables. Correlations were assessed using nonparametric Spearman ρ values. All density measures (ring-stage parasite density, gametocyte density and marker transcript density) were log10 transformed and Gaussian distributions with an identity link were used for the generalized additive mixed models.

The association between genetic marker transcript levels at timepoint T and total ring-stage density 14 days later (T^+14^) was determined by generalized additive mixed models for the five markers separately or in combination, whilst accounting for the influence of time and concurrent ring-stage and gametocyte densities and participant age (in categories); correlations between observations from the same individual were accounted for by a participant-specific random effect. A similar model was used to determine the association between marker transcript levels and gametocyte densities 14 days later whilst accounting for concurrent ring-stage and gametocyte densities. In an additional model, we tested the effect of marker transcript levels separately whilst accounting for concurrent transcripts of *uce* and concurrent gametocyte density.

The influence of mosquito exposure on marker transcripts on day 20 was assessed using a generalized additive model with an interaction term between mosquito exposure and marker transcripts on day 14 whilst accounting for concurrent ring-stage parasites and gametocytes on day 14, and age (in categories). To determine whether the association between day 14 ring-stage parasites and day 28 mature gametocytes differed between the mosquito exposed and control cohort, we used a similar generalized additive model with an interaction term between mosquito exposure and ring-stage parasites on day 14. The estimate (β) of the association between predictor and outcome variable was used to calculate the fold change (10^β^). Fold change can be interpreted as follows: when a predictor variable increases 10-fold, the outcome variable changes by a fold change of 10^β^. In models that shared an outcome variable, we accounted for multiple testing using the Benjamini Hochberg approach. Statistical analyses were performed using R software, version 2022.02.01. Full details are shown in the appendix.

### Role of funders

The funders had no role in study design, data collection, data analysis, interpretation of findings, or writing of the report.

## Results

A total of 11 genetic markers were selected from previous literature based on their association with gametocyte commitment or early gametocyte formation (Supplementary Table S3). These markers were initially tested on *P. falciparum* culture material to exclude those without detectable transcripts in asexual stages and to assess background levels in gametocytes. All 11 markers had detectable transcripts in asexual parasite stages (Supplementary Table S4). Compared to *ap2-g,* all markers except *msrp1, surfin1.2, surfin13.1, gexp-2, sir2a* had higher background transcript levels in mature gametocytes.

To down-select markers for a full evaluation, all 11 markers were further tested in a subset of samples (n = 7/476, 1.5%) from the cohort in Burkina Faso. Samples with distinct densities of asexual parasites (*sbp-1* transcripts) and mature gametocytes (*CCp4* plus *PfMGET* transcripts) were selected as highly informative to explore marker performance. Based on these analyses (Supplementary Table S5), six markers were excluded from further analyses since a background signal was present in parasite negative samples (n = 2 markers), in pure gametocytes (n = 2), or both (n = 2) (Supplementary Figure S1). The remaining five genetic markers were considered promising since they had detectable transcript levels in at least one ring-stage positive, gametocyte negative sample with detectable gametocytes 14 days later (Supplementary Table S5). Based on these results, we selected five promising genetic markers: *ap2-g*, *gexp-2*, *sir2a*, *surfin1.2*, *surfin13.1* for more extensive testing in the complete sample sets.

### Predicting gametocyte formation in controlled malaria infections

**Fig. 1:**
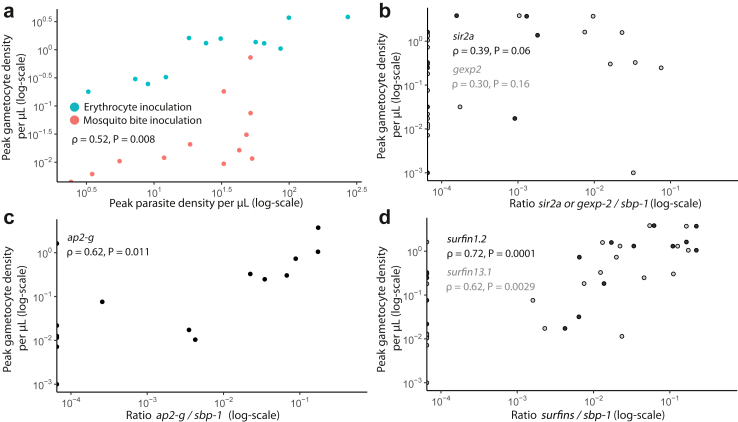
Peak parasite density and genetic marker transcripts in relation to subsequent peak gametocyte density upon controlled human malaria infection of naïve individuals. a) Peak parasite density measured by 18S qPCR in relation to peak gametocyte density measured by qRT-PCR. Colous indicate method of inoculation used, either through infected erythrocytes (erythrocyte inoculation) or through infectious mosquito bites (mosquito bite inoculation). b-d) The expression of a marker relative to *sbp-1* was determined by quantitative reverse transcriptase PCR (qRT-PCR) at the moment of treatment when all individuals were positive for *sbp-1*. At treatment, prevalence of *gexp-2* and *sir2a* transcripts was lowest: 16.7% (4/24) and 37.5% (9/24), respectively. Prevalence of *ap2-g, surfin1.2, surfin13.1* transcripts was 62.5% (10/16), 92.3% (12/23) and 71.4% (15/21), respectively. Data points from the one individual that did not develop detectable gametocytes are plotted at 10^−3^ gametocytes/μL (Log-transformation) and observations with no detectable transcripts for the indicated marker are depicted at 10^−4^ marker/*sbp-1* ratio. The relation between peak parasite density or marker transcript density and peak gametocyte density was assessed using Spearman rank correlation amongst all observations.

The five selected markers were then tested on samples from 24 healthy adults participating in a CHMI study^43^ (Supplementary Table S6); this sample set was selected since it had uniquely detailed longitudinal sample collection in a population that was free of gametocytes at the moment treatment was initiated to clear asexual parasites. All individuals had detectable ring-stage parasites at the time of treatment and 95.8% (23/24) had detectable gametocytes 5–16 days after treatment. The route of inoculation did not have a significant effect on the relation between peak asexual parasite density and peak gametocyte density^43^; both inoculation groups were pooled for genetic marker assessments. Peak asexual parasite density was positively correlated with peak gametocyte density (ρ = 0.52, P = 0.008 Spearman rank, Fig. 1a^43^). Peak gametocyte density followed peak asexual density after a median of 13.6 days (CI 12.3–14.8 days) and did not differ significantly between female (median 0.30 gametocytes/μL, IQR 0.05–1.33) and male participants (median 0.03 gametocytes/μL, IQR 0.01–0.32, P = 0.26 Mann Whitney U).

Transcripts of *gexp-2* and *sir2a* had the lowest prevalence of all five markers and were detected in 16.7% (4/24) and 37.5% (9/24) of study participants at the time of treatment, respectively. Prevalence of *ap2-g, surfin1.2, surfin13.1* transcripts was 62.5% (10/16), 92.3% (12/23) and 71.4% (15/21), respectively. Transcript levels of *sir2a* and *gexp-2* relative to *sbp-1* (i.e. transcripts per ring-stage parasite) correlated to subsequent peak gametocyte density, although these correlations were statistically not significant (*sir2a*: ρ = 0.39, P = 0.06, *gexp-*2: ρ = 0.30, P = 0.16, Spearman rank, Fig. 1b). The ratio of *ap2-g* to s*bp-1* transcripts at treatment correlated with subsequent peak gametocyte density (ρ = 0.62, P = 0.011, Spearman rank, Fig. 1c and^43^). The ratio of *surfin1.2* or *surfin13.1* to *sbp-1* transcripts at treatment was also positively correlated with subsequent peak gametocyte density, with *surfin1.2* showing the strongest correlation (ρ = 0.72, P = 0.0001, Spearman rank, Fig. 1d).

### Gametocyte carriage and parasite kinetics in naturally acquired infections

We further tested all five genetic markers in a longitudinal observational study in Burkina Faso. A total of 120 asymptomatically infected individuals (Supplementary Table S7) were recruited and monitored without antimalarial treatment for a maximum of 28 days. One individual had no detectable ring-stage parasites or gametocytes throughout the study period and was removed from the analyses (Supplementary Figure S2). None of the individuals developed symptoms during their follow-up. Plasma levels of CRP were measured as an estimate of inflammatory status; median CRP levels were 3.12 mg/L (IQR: 1.27–8.46) and 23.5% (28/119) of individuals had CRP levels above 10 mg/L, which is indicative of an acute phase response. Serological responses against a panel of three antigens related to cumulative exposure (AMA-1, MSP-1.19, GLURP.R2) and three antigens related to exposure in past six months (Rh2.2030, GEXP18, Etramp5.Ag1) were determined at baseline. All individuals had detectable antibody levels against all six antigens; the intensity of response varied between study participants and between antigens with AMA-1 and GLURP.R2 showing the highest responses (Supplementary Figure S3).

**Fig. 2:**
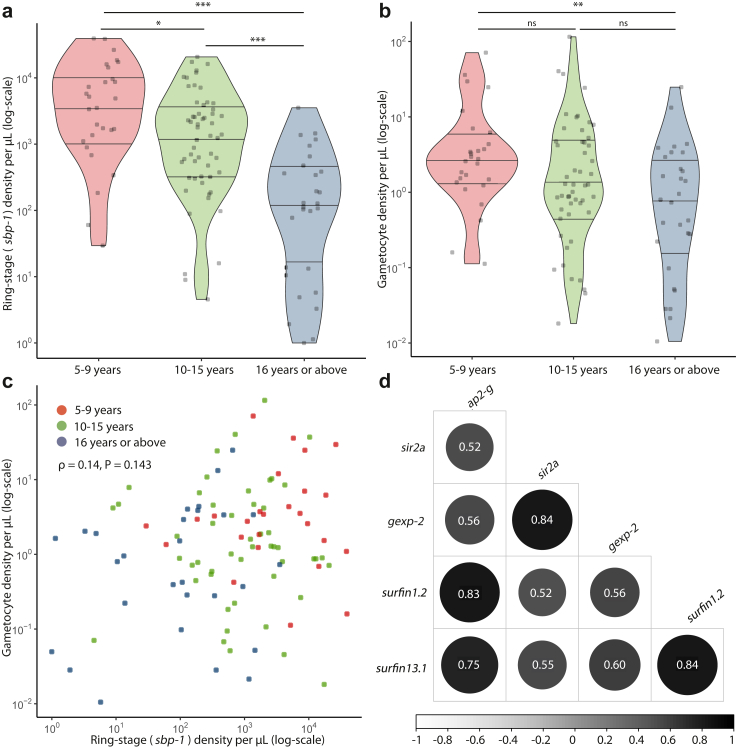
Ring-stage parasite and gametocyte densities at enrolment in the longitudinal follow-up study in Burkina Faso. a) Ring-stage parasite density (*sbp-1*) among qRT-PCR positive infections (n = 112) at enrolment, day 0, separated by age category. b) Gametocyte density among qRT-PCR positive infections at enrolment (n = 107) determined by qRT-PCR, separated by age category. Lines indicate median, upper- and lower-quartile densities. a,b) Amongst qRT-PCR positive observations, differences in ring-stage or gametocyte density between age categories were assessed using the Mann Whitney U test. A single asterisk indicates significance level of P < 0.05, double asterisks indicate significance level of P < 0.01, triple asterisks indicate significance level of P < 0.001. c) Ring-stage density at enrolment in relation to gametocyte density at enrolment for ring-stage parasite and gametocyte positive infections. Relation between ring-stage density and gametocyte density was assessed using spearman rank correlation using all observations at baseline (n = 115) d) Spearman correlation matrix for combinations of marker transcript levels at enrolment, including negative observations. Colour shade and circle size indicate the direction and strength of the correlation coefficient. A circle was only indicated when a Spearman rank correlation was statistically significant (P < 0.05).

At enrolment, ring-stage parasite and gametocyte prevalence by qRT-PCR was 97.4% (112/115) and 93.0% (107/115), respectively (Supplementary Table S8) and 43.4% (46/106) of infections were polyclonal as determined by *msp2* genotyping. Levels of the inflammatory marker CRP were positively correlated with ring-stage parasite density (ρ = 0.34, P = 0.0002, Spearman rank). Median concentration of CRP did not differ significantly between monoclonal (median 3.41 mg/L, IQR 1.15–14.8) and polyclonal infections (median 2.90 mg/L, IQR 1.46–8.80, P = 0.91, Mann Whitney U). Median ring-stage density was associated with age (Fig. 2a) but did not differ significantly between female (1364.7 parasites/μL, IQR 217.2–4066.3) and male (716.6 parasites/μL, IQR 187.5–3367.7, P = 0.649, Mann Whitney U) participants. There was no consistent pattern of marker transcripts relative to ring-stage parasites by age, except for *surfin1.2* and *surfin13.*1 that appeared highest in older individuals (Supplementary Figure S4).

Median gametocyte density among qRT-PCR positive enrolment samples was higher in participants aged 5–9 years (2.77 gametocytes/μL, IQR 1.42–5.28) compared to participants aged 16 years or above (0.80 gametocytes/μL, IQR 0.22–2.93, P = 0.005, Mann Whitney U) and participants aged 10–15 years (1.26 gametocytes/μL, IQR 0.57–4.68, P = 0.08, Mann Whitney U, Fig. 2b), although the latter was not statistically significant. Median gametocyte density did not differ significantly between female (1.69 gametocytes/μL, IQR 0.72–4.19) and male (1.28 gametocytes/μL, IQR 0.39–4.17, P = 0.456, Mann Whitney U) participants or between monoclonal (1.50 gametocytes/μL, IQR 0.35–3.95) and polyclonal infections (1.40 gametocytes/μL, IQR 0.43–4.30, P = 0.838, Mann Whitney U).

Ring-stage parasite density and mature gametocyte density amongst positive samples were positively correlated, but this observation was only statistically significant when combining all visits (ρ = 0.20, P < 0.0001, Spearman rank, Supplementary Figure S5), not when considering enrolment samples only (ρ = 0.14, P = 0.143, Spearman rank, Fig. 2c). At enrolment, transcript levels (transcripts/μL) of all genetic markers were statistically significantly correlated with each-other (Fig. 2d). There was no evidence for a modifying effect of antibody responses against blood-stage malaria antigens in the association between ring-stage parasite density at baseline and 14 days later (Supplementary Table S9).

To assess associations with gametocyte production, allowing for the known 8.5–14 day period of sequestration,^57^ we determined the correlation between markers at given time-points (T) with parasite or gametocyte densities at subsequent visits 14 days later (T^+14^). Although it did not reach statistical significance, transcript levels of *sir2a* on timepoint T were negatively associated with ring-stage density on timepoint T^+14^ (fold change = 0.78, 95% CI = 0.47–1.30, P = 0.341, t-test, Table 1). For all markers, we observed statistically significant positive associations between transcript levels on timepoint T and mature gametocyte density on timepoint T^+14^, without evidence for an interaction between antibody responses against blood-stage malaria antigens and marker transcript levels. A 10-fold increase in *sir2a* transcripts on timepoint T was associated with a 1.65 fold increase in gametocyte density at timepoint T^+14^ (95% CI = 1.22–2.24, P = 0.004, t-test, Table 2). A 10-fold increase in *ap2-g* transcripts on timepoint T was associated with a 1.37 fold increase in gametocyte density at timepoint T^+14^ (95% CI = 1.22–2.24, P = 0.004, t-test). A 10-fold increase in *gexp-2, surfin1.2* or *surfin13.1* transcripts was associated with a 1.40 (95% CI = 1.11–1.76, P = 0.008, t-test), 1.26 (95% CI = 1.05–1.51, P = 0.018, t-test), or 1.27 (95% CI = 1.04–1.55, P = 0.018, t-test) fold increase in gametocyte density at timepoint T^+14^, respectively. When combinations of the five markers were tested, the individual effects of *ap2-g* and *sir2a* remained statistically significant (Supplementary Table S10). To explore the robustness of our findings, we used *uce* as alternative marker of total parasite density; *sbp-1* is specific to ring-stage parasites whilst *uce* is expressed in all parasite stages. Similar effects of individual marker transcripts on future gametocyte density were observed when, instead of ring-stage density, concurrent *uce* transcripts were used (Supplementary Table S11); *ap2-g, sir2a* and *gexp-2* remained strong predictors of gametocyte density at timepoint T^+14^.

**Table 1:** Present marker transcript density in relation to future ring-stage (*sbp-1*) parasite density.

Marker transcripts per μL on T	Ring-stage parasite density per μL on T^+14^			
	Fold change	95% CI	P-value	R^2^
*ap2-g*	1.04	0.77–1.41	0.787	0.516
*sir2a*	0.78	0.47–1.30	0.341	0.542
*gexp-2*	0.98	0.65–1.46	0.914	0.474
*surfin1.2*	1.13	0.84–1.52	0.435	0.505
*surfin13.1*	0.99	0.72–1.36	0.926	0.523

To assess whether marker transcript density at timepoint T relates to ring-stage parasites 14 days later (T^+14^), a generalized additive model was used for five genetic markers separately with marker density at T as a predictor variable and parasite density at T^+14^ as an outcome variable. The model accounted for concurrent ring-stage parasites and gametocytes and included a random person effect to account for multiple observations from the same individual. An additional effect of age category and time was also accounted for. Fold change indicate the change in outcome variable when predictor variable increased 10-fold, 95% Confidence Interval (CI) indicates the ranges of fold changes per marker predictor variable. P-values below 0.05 were considered significant and R^2^ indicates the goodness of fit.

**Table 2:** Present marker transcript density in relation to future gametocyte density.

Marker transcripts per μL on T	Gametocyte density per μL on T^+14^			
	Fold change	95% CI	P-value	R^2^
*ap2-g*	1.37	1.14–1.64	0.004	0.760
*sir2a*	1.65	1.22–2.24	0.004	0.486
*gexp-2*	1.40	1.11–1.76	0.008	0.473
*surfin1.2*	1.26	1.05–1.51	0.018	0.454
*surfin13.1*	1.27	1.04–1.55	0.018	0.446

To assess whether marker transcript density at timepoint T relates to gametocyte densities 14 days later (T^+14^), a similar generalized additive model as described in Table 1 was used. The model also accounted for present ring-stage parasites, gametocytes, age-category, and included a random person effect. Fold change indicate the change in outcome variable when predictor variable increased 10-fold, 95% Confidence Interval (CI) indicates the ranges of fold changes per marker predictor variable. P-values below 0.05 were considered significant and R^2^ indicates the goodness of fit.

### Gametocyte production and infectivity after the exposure to uninfected mosquito bites

**Fig. 3:**
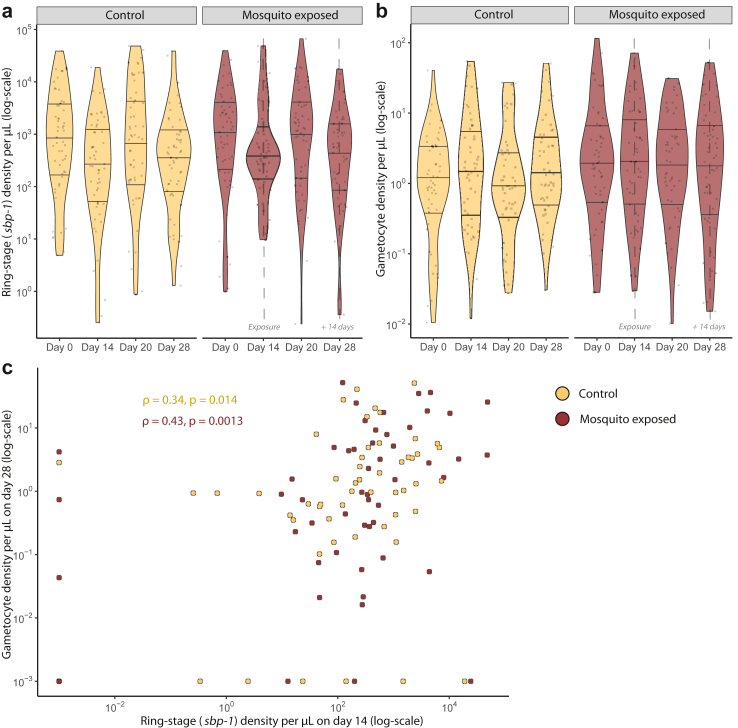
Parasite characteristics in relation to mosquito exposure in the longitudinal follow-up study. Ring-stage parasite density (a) and gametocyte density (b) separated by timepoint and exposure status. Lines in the violin plots indicate the median and upper- and lower-quartiles amongst *sbp-1* (n = 431) (a) and gametocyte (n = 417) (b) positive observations. a) Initial ring-stage parasite density on day 14 did not differ significantly between the mosquito-exposed (median 355.9 ring-stage parasites/μL, IQR 156.7–1060.6, n = 49) and control cohort (248.1 ring-stage parasites/μL, IQR 49.1–1398.3, P = 0.217, n = 53, Mann Whitney U). b) Initial gametocyte density on day 14 did not differ significantly between mosquito-exposed (2.05 gametocytes/μL, IQR 0.60–10.4, n = 51) and control cohort (1.64 gametocytes/μL, IQR 0.34–6.23, P = 0.466, n = 51, Mann Whitney U). Timing of skin-feeding (‘Exposure’) is indicated by a dashed line for the exposed cohort. On day 28, 14 days post skin feeding, median gametocyte density in the mosquito-exposed cohort did not differ significantly from the control cohort (P = 0.871, Mann Whitney U). c) The relation between ring-stage parasite density at the moment of mosquito exposure, on day 14, and future gametocyte density on day 28. Spearman rho indicates the correlation between ring-stage parasite density and gametocyte density for the two cohort types separately.

On day 14 of the follow-up, 49.2% of the cohort (n = 59) participated in direct skin feeding assays, exposing them to bites of 60 uninfected mosquitoes (‘mosquito exposed cohort’). No mosquito skin feeding assays were performed on the other 60 participants (‘control cohort’). Sampling of wild-caught mosquitoes in participant households confirmed negligible natural exposure during the study period in the dry season (Supplemental Data). On the time-point immediately before controlled mosquito-exposure (day 14 after study inclusion); median ring-stage parasite density did not differ between the mosquito-exposed (355.9 ring-stage parasites/μL, IQR 156.7–1060.6) and control cohort (248.1 ring-stage parasites/μL, IQR 49.1–1398.3, P = 0.217, Mann Whitney U, Fig. 3a). Median gametocyte density on day 28 was 2.02 gametocytes/μL (IQR 0.32–5.78) in the mosquito-exposed cohort and 1.35 gametocytes/μL (IQR 0.59–4.66) in the control cohort (P = 0.871, Mann Whitney U, Fig. 3b). Ring-stage parasite density on day 14 was positively associated with gametocyte density on day 28 for both the control (ρ = 0.34, P = 0.014, Spearman rank) and mosquito-exposed (ρ = 0.43, P = 0.0013, Spearman rank) cohorts (Fig. 3c) without evidence for a modulating effect of mosquito exposure (P = 0.379, t-test, Supplementary Table S12). We observed no evidence for an effect of experimental mosquito exposure on marker transcription (Supplementary Table S13). To explore if experimental mosquito exposure induced a host response to mosquito salivary antigens, anti-gSG6 antibodies were measured at day 14 (baseline) and day 28 (14 days post exposure). Whilst median antibody responses to the mosquito saliva antigen gSG6 increased with age (Supplementary Figure S6), anti-gSG6 antibodies generally declined during follow-up (day 28 versus day 14) for both the control (Median Optical Density day 14 = 0.10, day 28 = 0.08, P = 0.218, Mann Whitney U) and mosquito exposed cohort (Median Optical Density day 14 = 0.10, day 28 = 0.09, P = 0.258, Mann Whitney U), although these differences were not statistically significant (Supplementary Figure S6).

In this cohort of individuals with chronic low-density *P. falciparum* infections, 119 membrane feeding experiments were conducted on the entire cohort on day 14 and 117 on day 28. Skin feeding experiments were performed on 59 individuals on day 14. Out of a total of 295 feeding experiments conducted, only 8.8% (16/295) resulted in infected mosquitoes. Eleven individuals infected mosquitoes in at least one feeding experiment, five of those individuals infected mosquitoes on day 28. The proportion of infectious individuals on day 28 was similar among individuals who were exposed to mosquito bites 14 days prior (3.39%, 2/59 individuals; 53.3%, 16/30 infected mosquitoes) compared to those who were not (5.00%, 3/60 individuals; 46.7%, 14/30 infected mosquitoes) (Supplementary Table S14).

## Discussion

Whilst circulating in the human bloodstream, *Plasmodium* parasites need to balance asexual parasite multiplication that ensures survival in the human host and gametocyte formation that allows onward transmission and long-term survival. Despite ongoing efforts to disrupt malaria transmission and the pivotal role that gametocytes play in this process, our understanding of the factors governing gametocyte commitment remains incomplete. The current study assessed the predictive value of putative markers of parasite dynamics in two malaria-infected populations and identified *ap2-g* and *sir2a* as particularly informative of gametocyte productivity. Using these markers, we observed no evidence that experimental exposure to *Anopheles* mosquitoes stimulates gametocyte formation in asymptomatic natural infections.

Previous studies that were largely reliant on in vitro experiments have identified several genetic markers upregulated in parasites with relatively high gametocyte conversion rates.^4^^,^^6^^,^^24^^,^^34^ A handful of markers were further shown to be associated with ex vivo gametocyte formation when donor blood was used to quantify gametocyte formation in short-term culture, thereby directly capturing the committed parasite fraction.^4^^,^^24^ However, very few studies have tested the performance of a subset of these genetic markers in a longitudinal framework^43^^,^^58^ and only one tested their performance in asymptomatic individuals without interference of anti-malarial drug exposure.^7^ Here, we performed the first study to directly compare the performance of 11 genetic markers that were previously identified for potential linkage to gametocyte commitment. Based on their performance on parasite culture material and a small set of field samples, we prioritized five genetic markers (*sir2a, ap2-g, gexp02, surfin1.2, surfin13.1*) for extensive evaluation in two malaria-infected cohorts.

Our findings demonstrate that three (*ap2-g, surfin1.2, surfin13.*1) markers associated with peak gametocytaemia in experimentally infected individuals and all five markers positively associated with gametocyte formation in naturally infected asymptomatic parasite carriers in Burkina Faso. Expression of genetic markers occurs as early as the sexually-committed ring stage^24^^,^^34^^,^^51^ or, in the case of *sir2a*, the asexual ring stage.^41^ The predictive value of *gexp-2, sir2a* and *ap2-g* for future gametocyte formation appeared strongest; when examining combinations of markers, only *sir2a* remained a consistent significant predictor of future gametocyte densities. *sir*2a was first described as an epigenetic factor involved in transcriptional control of *var* virulence genes in *P. falciparum*.^59^^,^^60^ *Sir2a* mutant parasites display enhanced rRNA transcription associated with increased parasite proliferation^41^ and *sir2a* levels appear to be lower in afebrile versus febrile infections.^61^ Interestingly, although not statistically significant, the effect of *sir2a* transcripts on asexual multiplication was negative and *sir2a* was significantly predictive of gametocyte formation in our longitudinal study. This corroborates findings of a recent cross-sectional study where expression of *sir2a* was negatively correlated to parasite biomass and positively correlated to *ap2-g* expression.^13^ These findings are also in line with a previous epidemiological study suggesting that parasite populations with low investment in asexual multiplication display highest investment in gametocyte formation.^7^

Using our genetic markers, we tested the long-standing hypothesis that exposure to mosquito bites may stimulate enhanced gametocyte formation. From an evolutionary perspective, it could be beneficial for parasites to balance asexual multiplication and gametocyte formation based on the presence or absence of suitable vectors, as is observed in other parasite systems.^14^ For malaria, the hypothesis of mosquito probing inducing gametocytogenesis received indirect support from epidemiological studies reporting increased gametocyte densities at the beginning of transmission season, before new infections were acquired.^22^^,^^23^ In our current study, we did not observe a relationship between controlled exposure to *Anopheles gambiae* mosquitoes and gametocyte formation; exposure to 60 uninfected *Anopheles* bites at a single time-point did not increase investment in gametocyte formation compared to control individuals who were not experimentally exposed to mosquito bites. Moreover, anti-gSG6 antibodies did not differ significantly between exposed and control individuals, whereas other studies described quantifiable gSG6 antibody responses after natural mosquito exposure.^62^ Whilst our data argue against a strong and rapid impact of mosquito exposure on gametocyte formation, we cannot rule out an impact of more natural exposure to mosquitoes, where biting exposure (and immune insult) may be less intense but more repetitive and prolonged.

In conclusion, we confirm the in vivo predictive value of five molecular markers for gametocyte formation in naturally infected asymptomatic parasite carriers and report evidence that quantification of *sir2a* and *ap2-g* transcripts may be particularly useful in improving our understanding of the balance between asexual multiplication and gametocyte formation in natural infections. We further report that a single high intensity mosquito biting event appears not to increase gametocyte commitment rates in naturally infected individuals, which does not support the hypothesis that gametocyte conversion is influenced by vector exposure.

## Contributors

A.T. , K.C., W.S., C.D., and T.B. designed the longitudinal study. A.B., M.G., A.O., I.S. and S.A.C were responsible for data collection in Burkina Faso. K.C and T.B. designed the controlled human malaria infection trial. K.L., K.T., W.G., E.D., and S.L.B. performed laboratory analyses. M.M. contributed to molecular analysis and interpretation of results. T.B., K.C., A.B., and S.L.B. have accessed and verified the data. S.L.B., J.R., and T.B. performed statistical analyses. The first draft of the manuscript was written by S.L.B., A.B., K.L., C.M.A and T.B. All authors contributed to the review and have red and approved of the final manuscript.

## Data sharing statement

The data that support the findings of this study are available in the supplementary material. All underlying data is available online (10.5061/dryad.w3r22810p and 10.5061/dryad.vq83bk3nq).

## Declaration of interests

We declare no competing interests.

## Appendix A Supplementary data

Supplementary Figure S1 Procedure of selecting potential genetic markers of gametocyte commitment. An initial selection of genetic markers (n = 11) was based on literature search and first tested on asexual stages and gametocytes of NF54 3D7 *Plasmodium falciparum* parasites. Marker transcript levels were assessed using quantitative reverse transcriptase PCR (qRT-PCR). All markers had detectable transcript levels in ring-stage parasites and were selected for further testing on a subset of field samples from Burkina Faso, including : i) samples negative for gametocytes and ring-stage parasites (n = 3), ii) samples with mature gametocytes only and no measured ring-stage parasites (n = 2), iii) ring-stage positive, gametocyte negative samples with detectable gametocytes 14 days later (‘clean commitment samples’, n = 2). Markers were excluded when a background signal in parasite (ring-stage and gametocyte) negative samples was detected. Markers were also excluded when a background signal was detected in both samples with mature gametocytes only. Final marker selection consisted of 5 markers: *ap2-g, gexp-2, sir2a, surfin1.2,* and *surfin13.1.*

Supplementary Figure S2 Longitudinal follow-up study procedures and sample processing. Recruited individuals from Burkina Faso aged 5 years or above and with asymptomatic malaria and a microscopic parasite density of at least 100 parasites/μL. Participants were asked to attend the clinic 14, 20, and 28 days post enrolment (day 0). On day 14, approximately half of the cohort was exposed to 60 uninfected *Anopheles gambiae* bites through direct skin-feeding. Number of participants is indicated by upper case (N), number of samples by lower case (n).

Supplementary Figure S3 Antibody responses against a panel of six *Plasmodium falciparum* blood-stage antigens detected at baseline in the longitudinal cohort study in Burkina Faso. Antibody responses were detected on a Luminex platform against three antigens associated to cumulative exposure (AMA-1, MSP-1.19, GLURP.R2) and to exposure in the past six months (GEXP18, Etramp5.Ag1, Rh2.2030). Antibody responses were detected in plasma samples of 119 individuals at baseline; at day 0 (n = 115) or day 14 (n = 4) of follow-up.

Supplementary Figure S4 The ratio of gametocyte or marker transcripts to ring-stage parasites at enrolment in the longitudinal cohort study in Burkina Faso. a) Gametocyte and ring-stage parasite (*sbp-1*) densities were quantified using qRT-PCR and the ratio of gametocyte to ring-stage parasites was plotted per age category amongst enrolment observations positive by qRT-PCR. Amongst qRT-PCR positive enrolment samples (n = 104), the median ratio gametocytes to ring-stage parasites (*sbp-1*) was significantly higher in individuals aged 16 years or above compared to individuals aged 10-15 years (P = 0.019, Mann Whitney U) and individuals aged 5-9 years (P = 0.009, Mann Whitney U). b-f) Marker transcript levels for five genetic markers were quantified using qRT-PCR. The expression of genetic markers relative to *sbp-1* was plotted per age category at the time of enrolment. For *surfin1.2* and *surfin13.1,* the median ratio of marker transcripts to *sbp-1* amongst qRT-PCR positive enrolment samples was highest in individuals aged 16 years or above compared to individuals aged 5-9 years (*surfin1.2*: P = 0.035, *surfin13.1*: P = 0.006, Mann Whitney U) and individuals aged 10-15 years (*surfin1.2*: P = 0.031, *surfin13.1*: P = 0.020, Mann Whitney U). Lines in the violin plots indicate the median and upper- and lower-quartiles amongst qRT-PCR positives. Differences in marker or gametocyte to *sbp-1* ratios per age category were determined using the Mann-Whitney U test, excluding negative observations.

Supplementary Figure S5 The relationship between ring-stage parasite density and gametocyte density in the longitudinal follow-up study. Ring-stage (*sbp-1*) parasite density in relation to gametocyte density of all ring-stage parasite and gametocyte positive visits, separated by age category. Spearman rho is indicated in text.

Supplementary Figure S6 Anti-gSG6 IgG responses upon exposure to mosquitoes in the longitudinal cohort study. At day 14 of the follow-up half of the Burkinabe cohort (n = 59) was exposed to 60 uninfected mosquito bites. Antibodies against gSG6 were measured at baseline (day 14) and two weeks post-exposure (day 28) using ELISA and expressed as 450nm OD-values. a) Anti-gSG6 antibodies per age category on day 14 and day 28 combined. Due to plasma availability, pair-wise comparisons of 73 individuals could be made; 18 individuals aged 5-9 years old, 36 individuals aged 10 to 15 years old, and 19 individuals aged 16 years or above. b) Anti-gSG6 responses from exposed (n=36 individuals) versus control individuals (n=37 individuals) on 14 and day 28. c) The relation between an individual’s anti-gSG6 antibodies at baseline (day 14) and 2 week later (day 28) for exposed versus control individuals. Spearman rho indicates the correlation between day 14 and day 28 anti-gSG6 antibodies for the two cohort types separately.

Supplementary Tables

Supplementary_Data_Ento

Appendix

Supplementary_Data_Primer_Efficiency
