# Supplementary material for: Plasmodium falciparum gametocyte production correlates with genetic markers of parasite replication but is not influenced by experimental exposure to mosquito biting
Source: eBioMedicine. 2024 Jun 19;105:105190. doi: 10.1016/j.ebiom.2024.105190 (PMC11239461; doi:10.1016/j.ebiom.2024.105190)
Supplement: Supplementary Figure S1 — Procedure of selecting potential genetic markers of gametocyte commitment. An initial selection of genetic markers (n = 11) was based on literature search and first tested on asexual stages and gametocytes of NF54 3D7 Plasmodium falciparum parasites. Marker transcript levels were assessed using quantitative reverse transcriptase PCR (qRT-PCR). All markers had detectable transcript levels in ring-stage parasites and were selected for further testing on a subset of field samples from Burkina Faso, including : i) samples negative for gametocytes and ring-stage parasites (n = 3), ii) samples with mature gametocytes only and no measured ring-stage parasites (n = 2), iii) ring-stage positive, gametocyte negative samples with detectable gametocytes 14 days later (‘clean commitment samples’, n = 2). Markers were excluded when a background signal in parasite (ring-stage and gametocyte) negative samples was detected. Markers were also excluded when a background signal was detected in both samples with mature gametocytes only. Final marker selection consisted of 5 markers: ap2-g, gexp-2, sir2a, surfin1.2, and surfin13.1. [file mmc1.pdf]

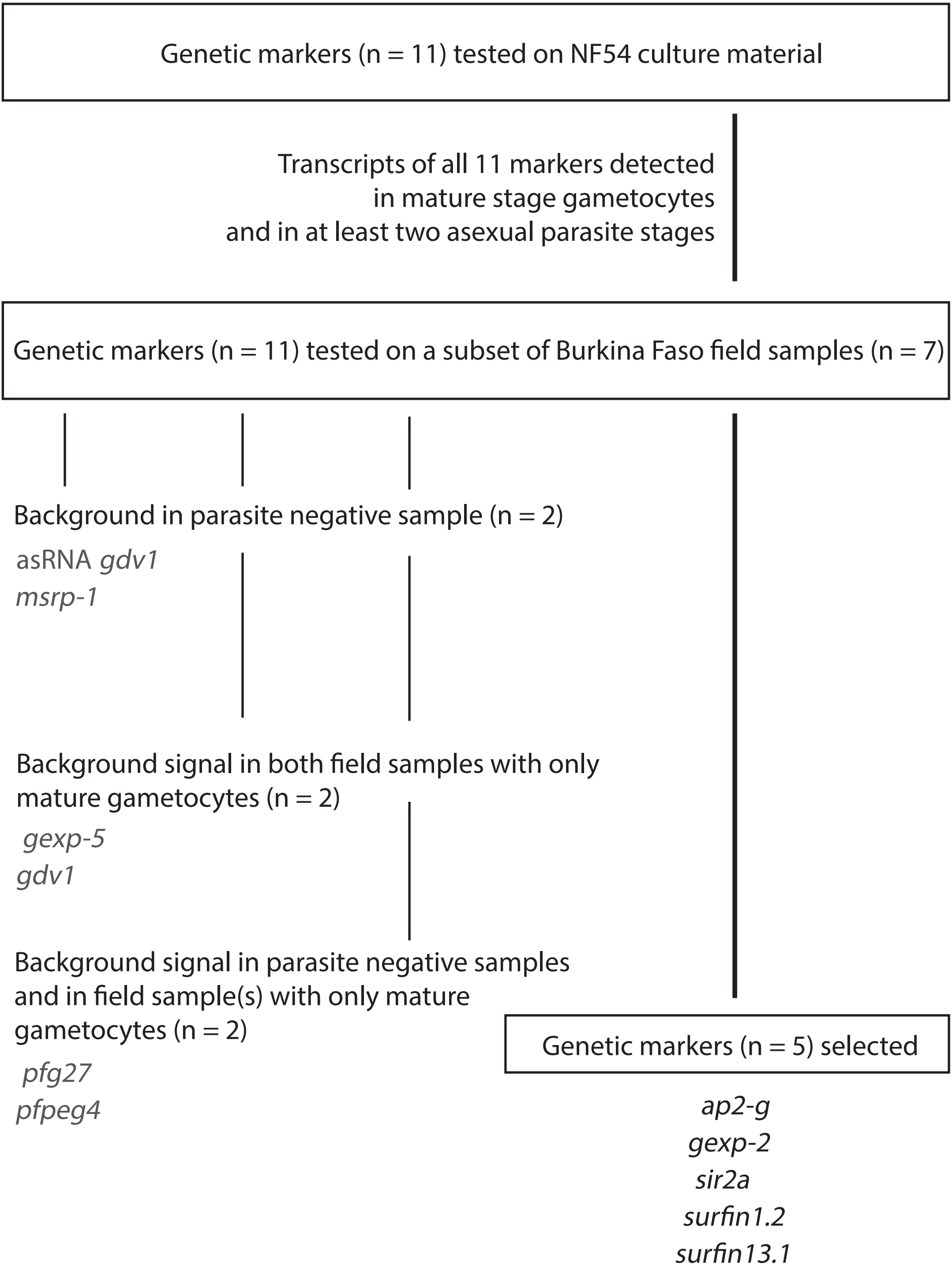
Genetic markers (n = 11) tested on NF54 culture material
Transcripts of all 11 markers detected in mature stage gametocytes and in at least two asexual parasite stages
Genetic markers (n = 11) tested on a subset of Burkina Faso field samples (n = 7)
Background in parasite negative sample (n = 2)
asRNA *gdv1*
msrp-1
Background signal in both field samples with only mature gametocytes (n = 2)
gexp-5
gdv1
Background signal in parasite negative samples and in field sample(s) with only mature gametocytes (n = 2)
pfg27
pfpeg4
Genetic markers (n = 5) selected
ap2-g
gexp-2
sir2a
surfin1.2
surfin13.1
