# Supplementary figures and images for: Plasmodium falciparum gametocyte production correlates with genetic markers of parasite replication but is not influenced by experimental exposure to mosquito biting
Source: eBioMedicine. 2024 Jun 19;105:105190. doi: 10.1016/j.ebiom.2024.105190 (PMC11239461; doi:10.1016/j.ebiom.2024.105190)

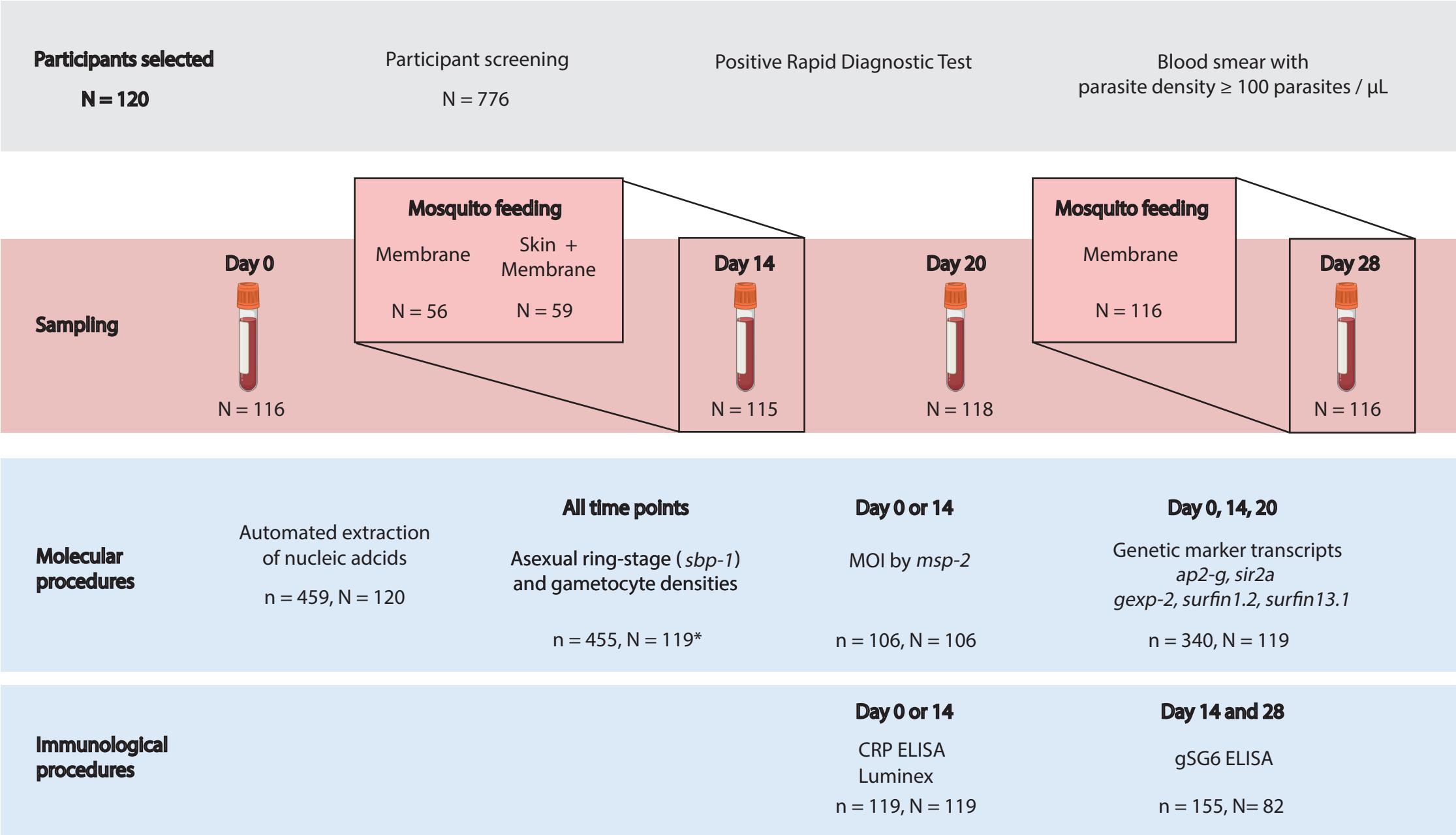

\*Exclusion of 1 participant without gametocytes or detectable ring-stages

Supplement: Supplementary Figure S2 — Longitudinal follow-up study procedures and sample processing. Recruited individuals from Burkina Faso aged 5 years or above and with asymptomatic malaria and a microscopic parasite density of at least 100 parasites/μL. Participants were asked to attend the clinic 14, 20, and 28 days post enrolment (day 0). On day 14, approximately half of the cohort was exposed to 60 uninfected Anopheles gambiae bites through direct skin-feeding. Number of participants is indicated by upper case (N), number of samples by lower case (n). [file mmc2.pdf]

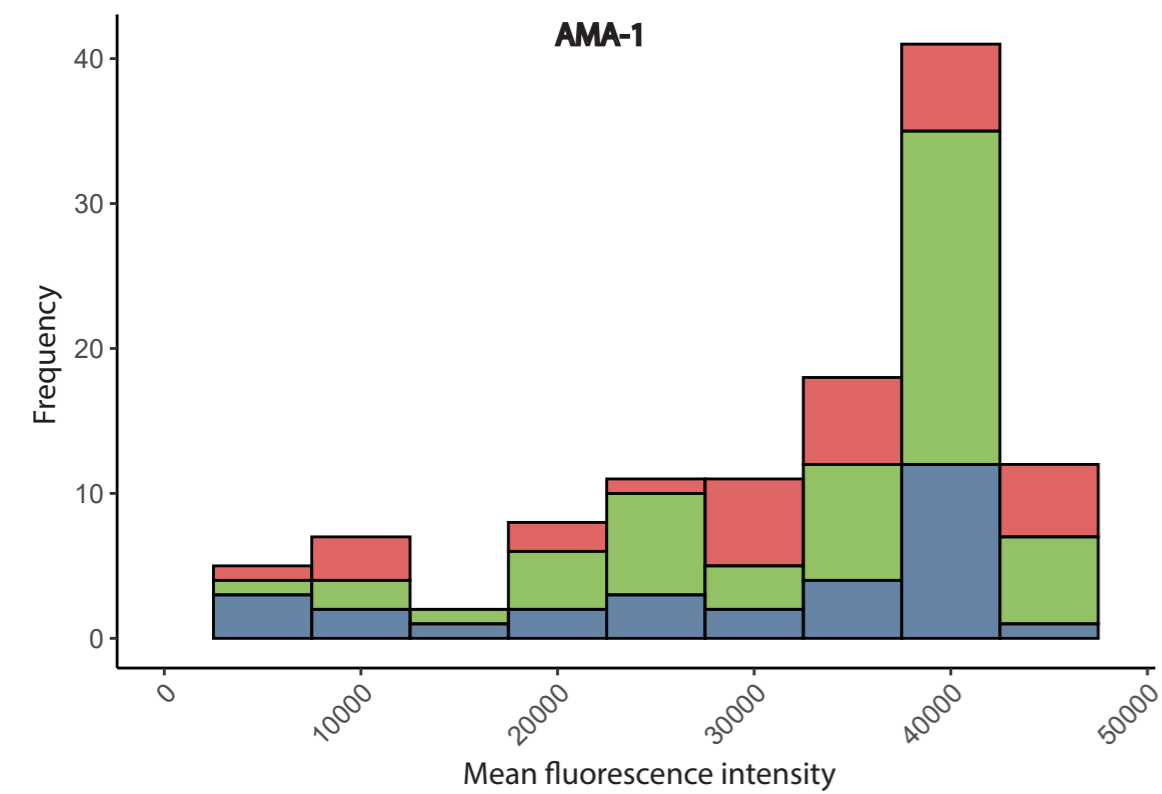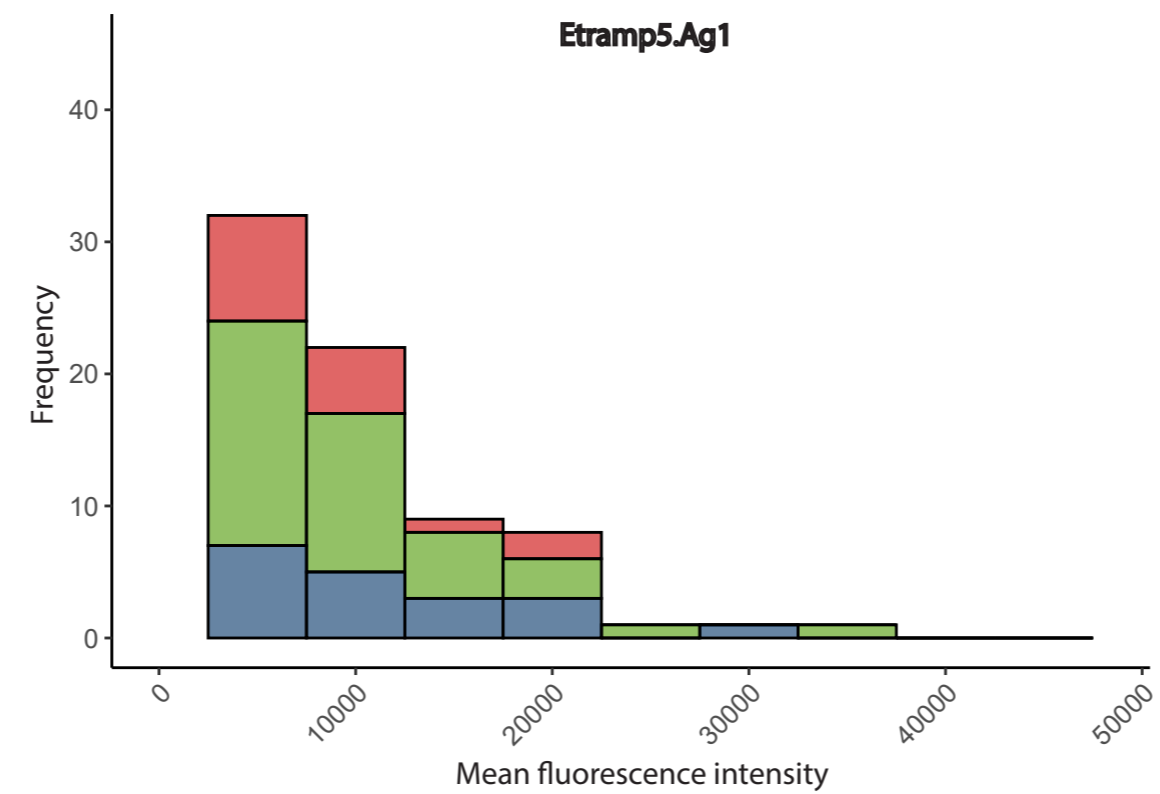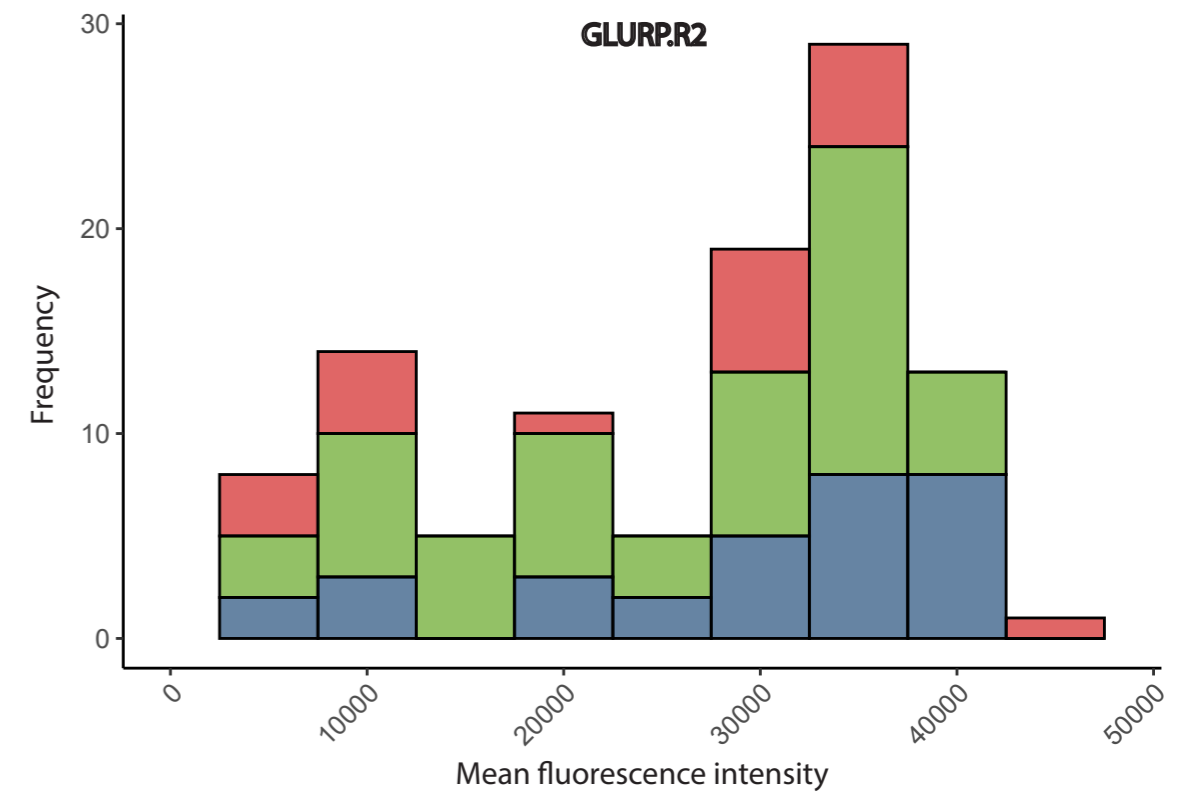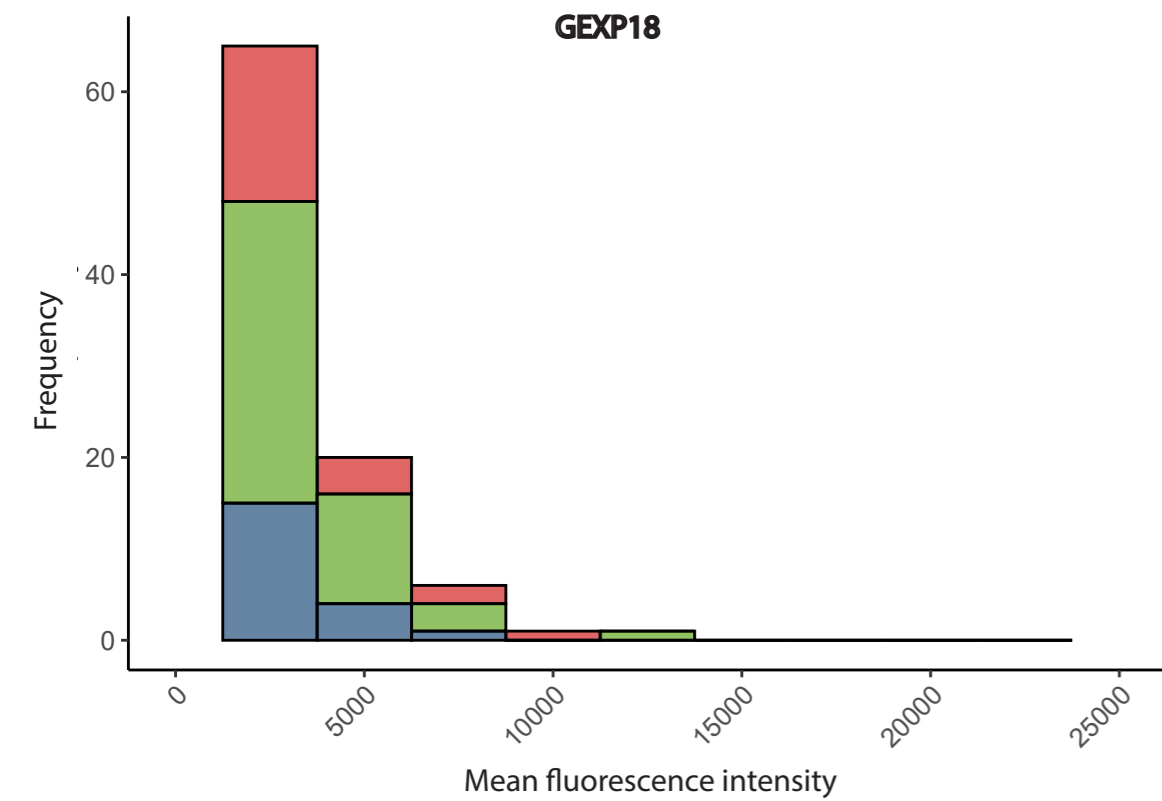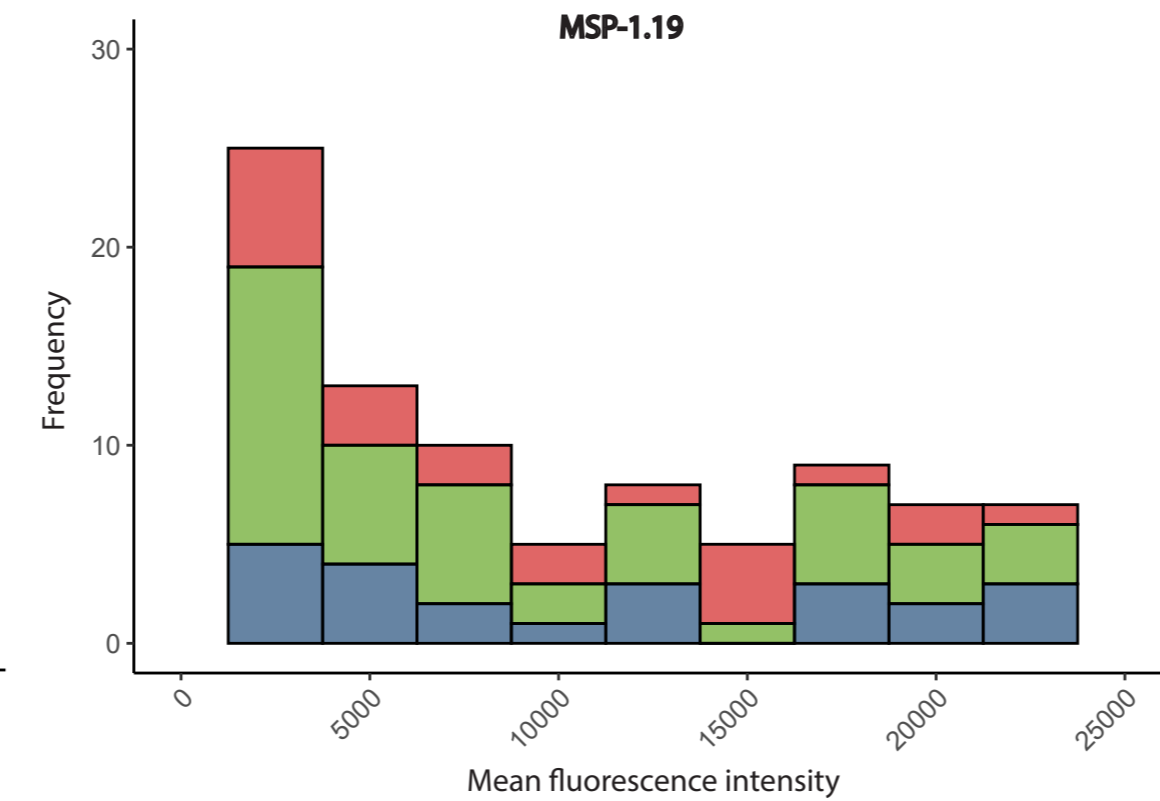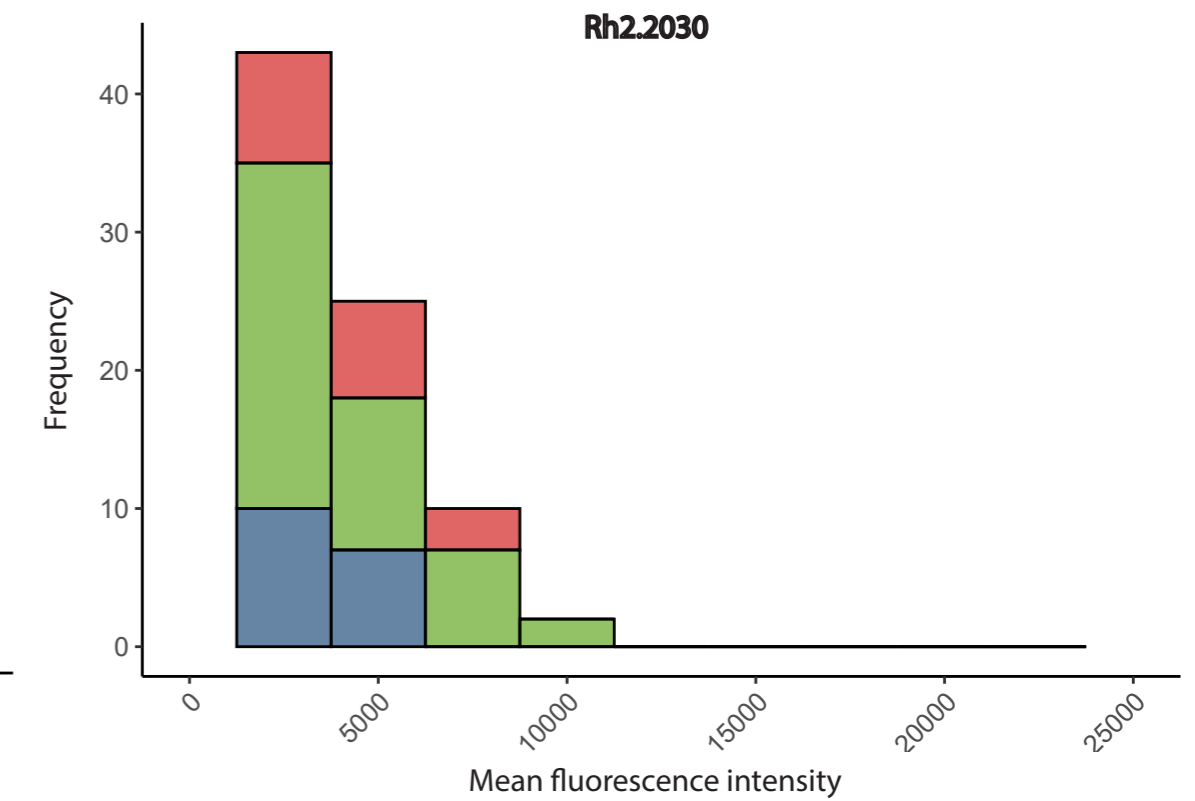

5 - 10 years old    10 - 15 years old    16 years or older

Supplement: Supplementary Figure S3 — Antibody responses against a panel of six Plasmodium falciparum blood-stage antigens detected at baseline in the longitudinal cohort study in Burkina Faso. Antibody responses were detected on a Luminex platform against three antigens associated to cumulative exposure (AMA-1, MSP-1.19, GLURP.R2) and to exposure in the past six months (GEXP18, Etramp5.Ag1, Rh2.2030). Antibody responses were detected in plasma samples of 119 individuals at baseline; at day 0 (n = 115) or day 14 (n = 4) of follow-up. [file mmc3.pdf]

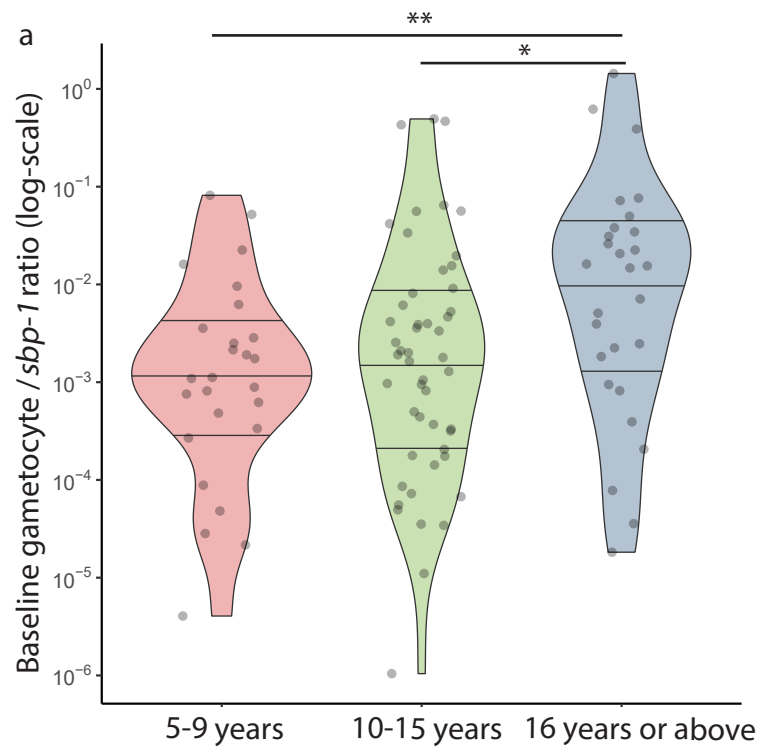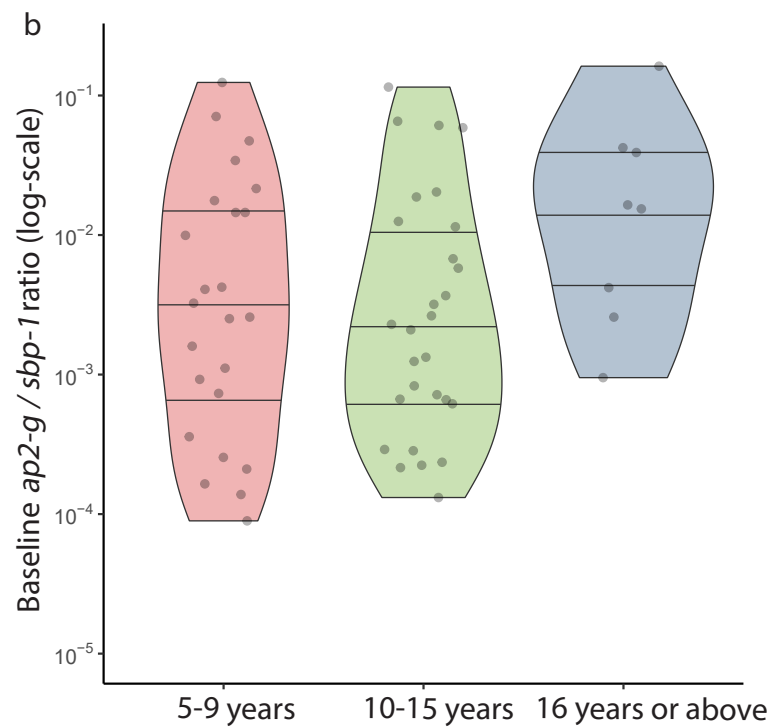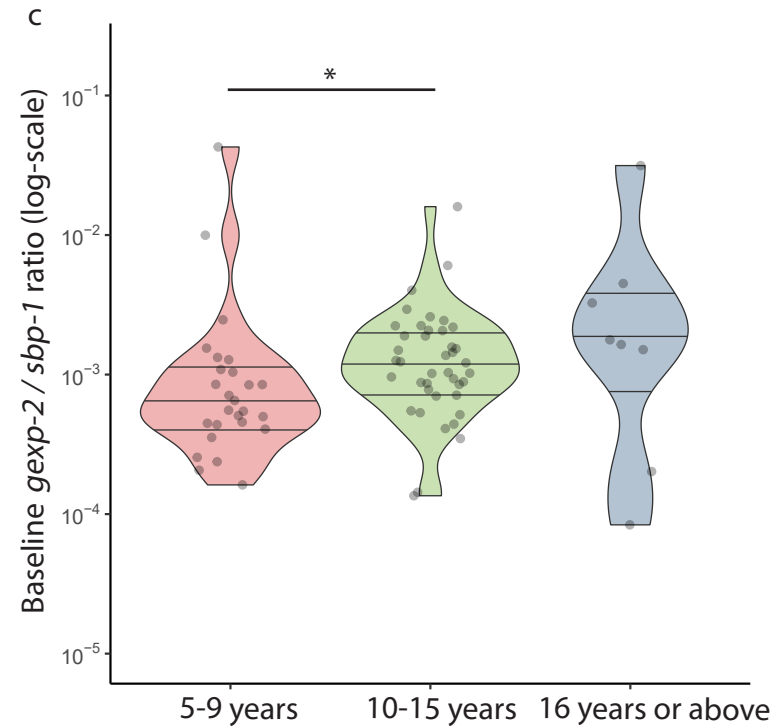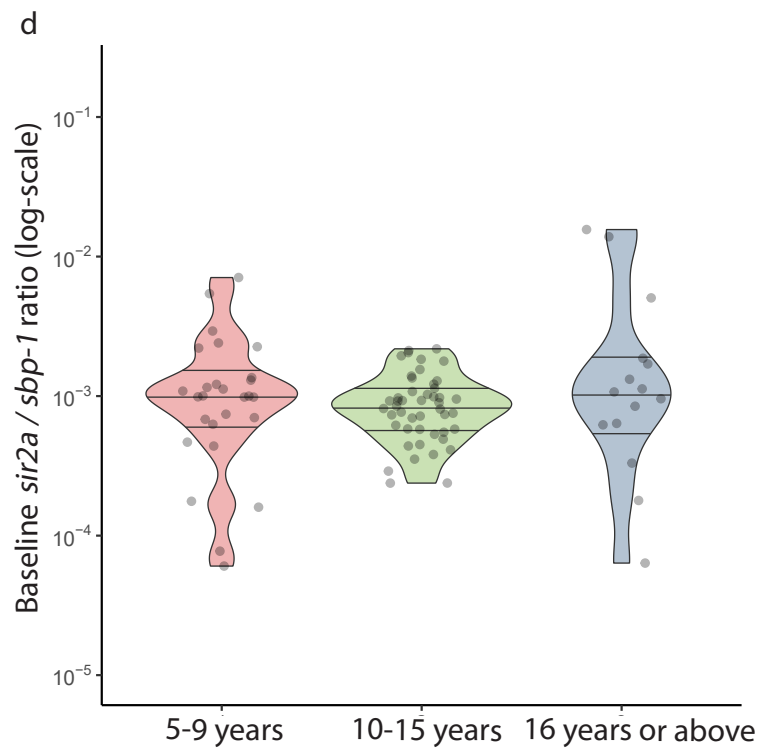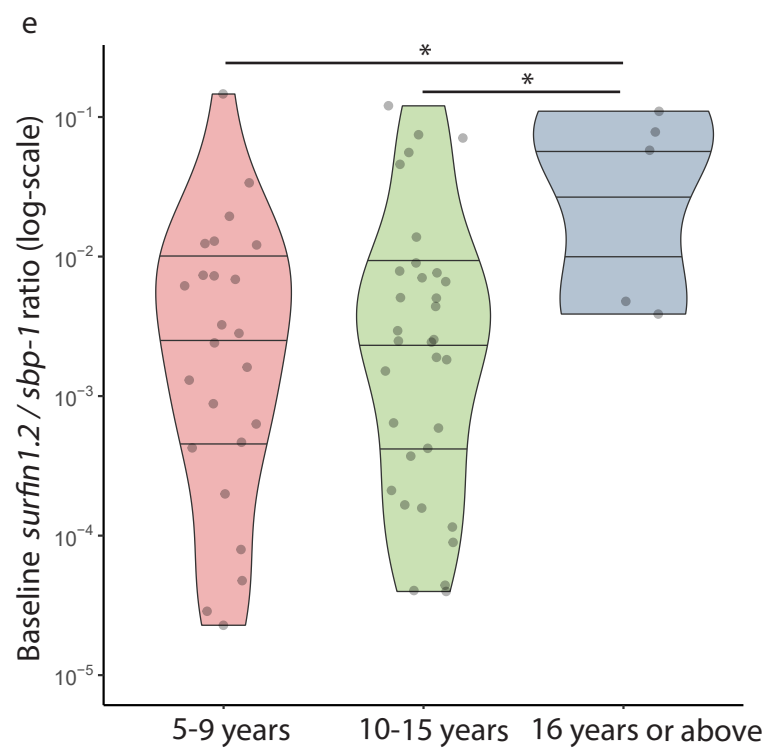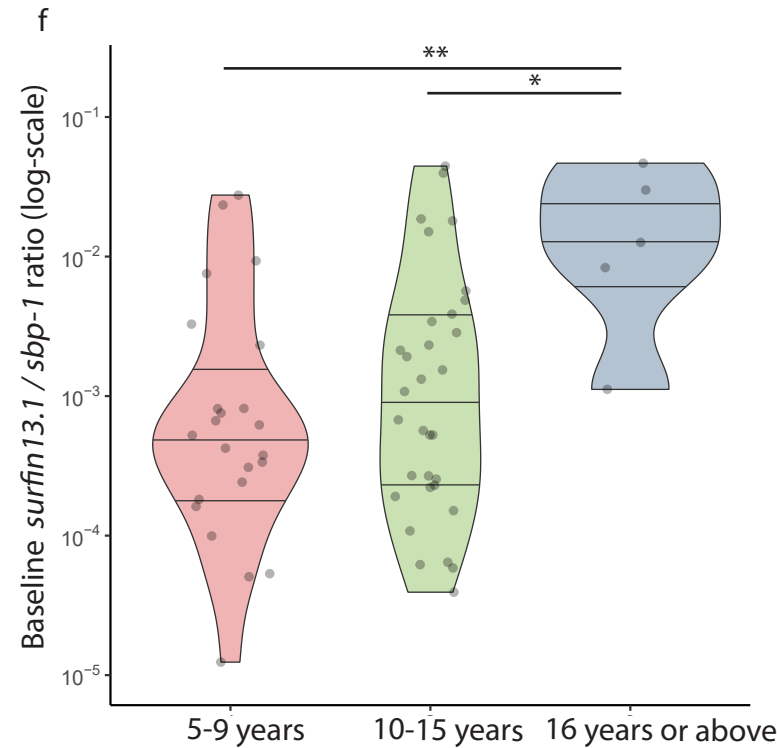

Supplement: Supplementary Figure S4 — The ratio of gametocyte or marker transcripts to ring-stage parasites at enrolment in the longitudinal cohort study in Burkina Faso. a) Gametocyte and ring-stage parasite (sbp-1) densities were quantified using qRT-PCR and the ratio of gametocyte to ring-stage parasites was plotted per age category amongst enrolment observations positive by qRT-PCR. Amongst qRT-PCR positive enrolment samples (n = 104), the median ratio gametocytes to ring-stage parasites (sbp-1) was significantly higher in individuals aged 16 years or above compared to individuals aged 10-15 years (P = 0.019, Mann Whitney U) and individuals aged 5-9 years (P = 0.009, Mann Whitney U). b-f) Marker transcript levels for five genetic markers were quantified using qRT-PCR. The expression of genetic markers relative to sbp-1 was plotted per age category at the time of enrolment. For surfin1.2 and surfin13.1, the median ratio of marker transcripts to sbp-1 amongst qRT-PCR positive enrolment samples was highest in individuals aged 16 years or above compared to individuals aged 5-9 years (surfin1.2: P = 0.035, surfin13.1: P = 0.006, Mann Whitney U) and individuals aged 10-15 years (surfin1.2: P = 0.031, surfin13.1: P = 0.020, Mann Whitney U). Lines in the violin plots indicate the median and upper- and lower-quartiles amongst qRT-PCR positives. Differences in marker or gametocyte to sbp-1 ratios per age category were determined using the Mann-Whitney U test, excluding negative observations. [file mmc4.pdf]

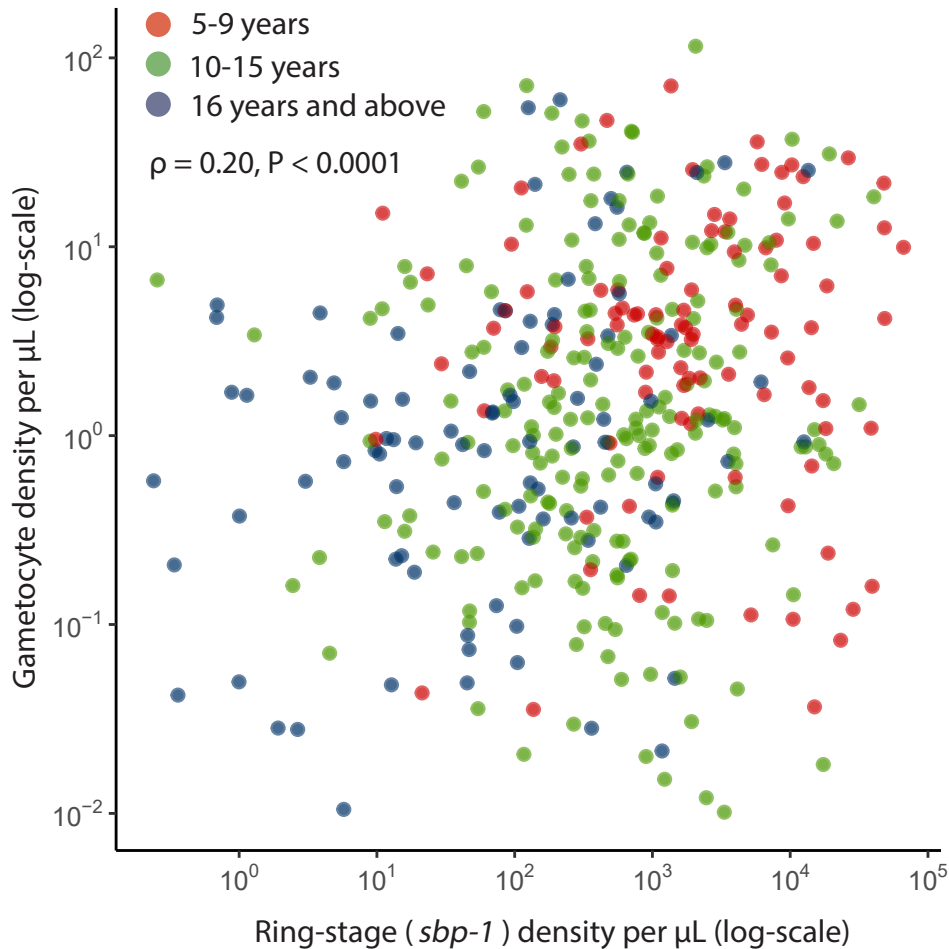

Supplement: Supplementary Figure S5 — The relationship between ring-stage parasite density and gametocyte density in the longitudinal follow-up study. Ring-stage (sbp-1) parasite density in relation to gametocyte density of all ring-stage parasite and gametocyte positive visits, separated by age category. Spearman rho is indicated in text. [file mmc5.pdf]

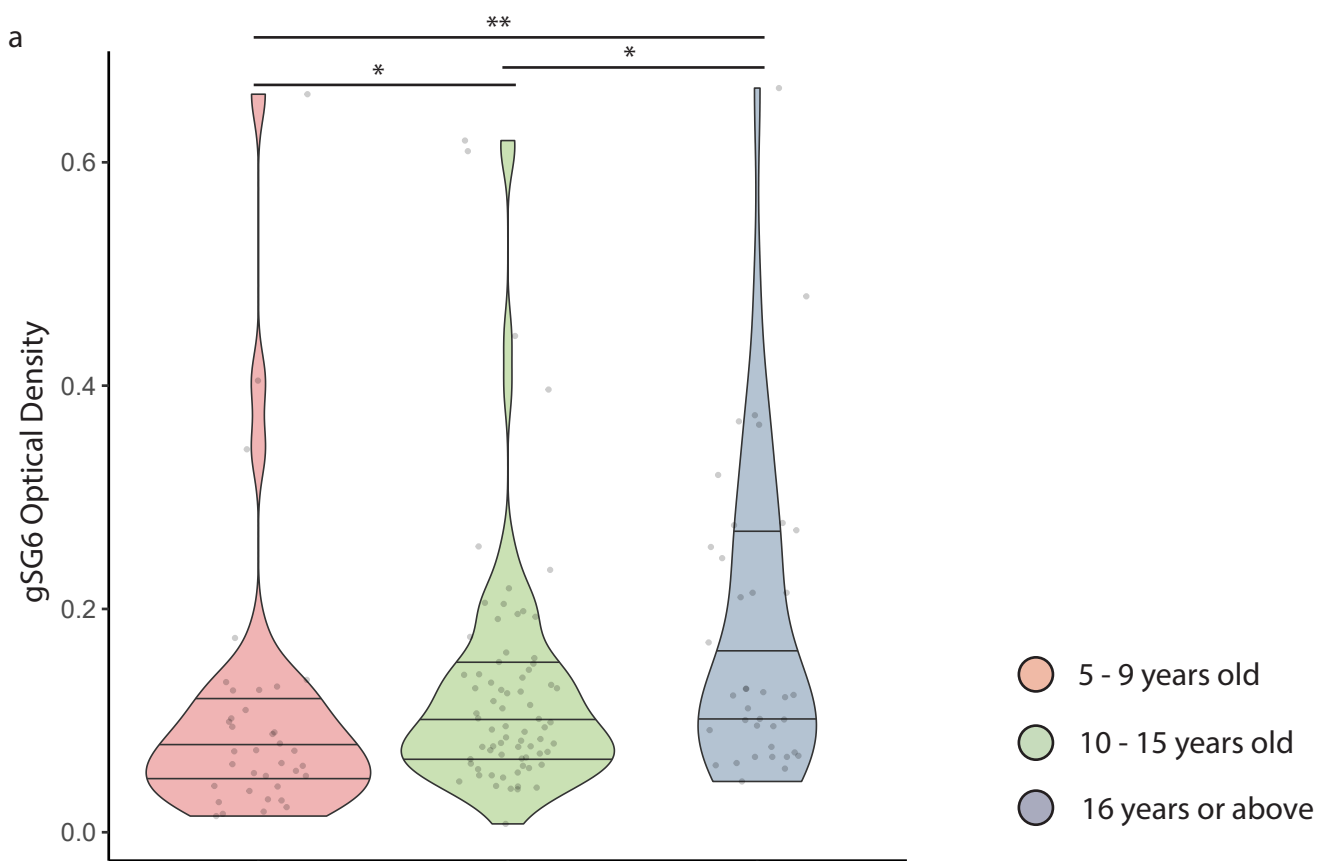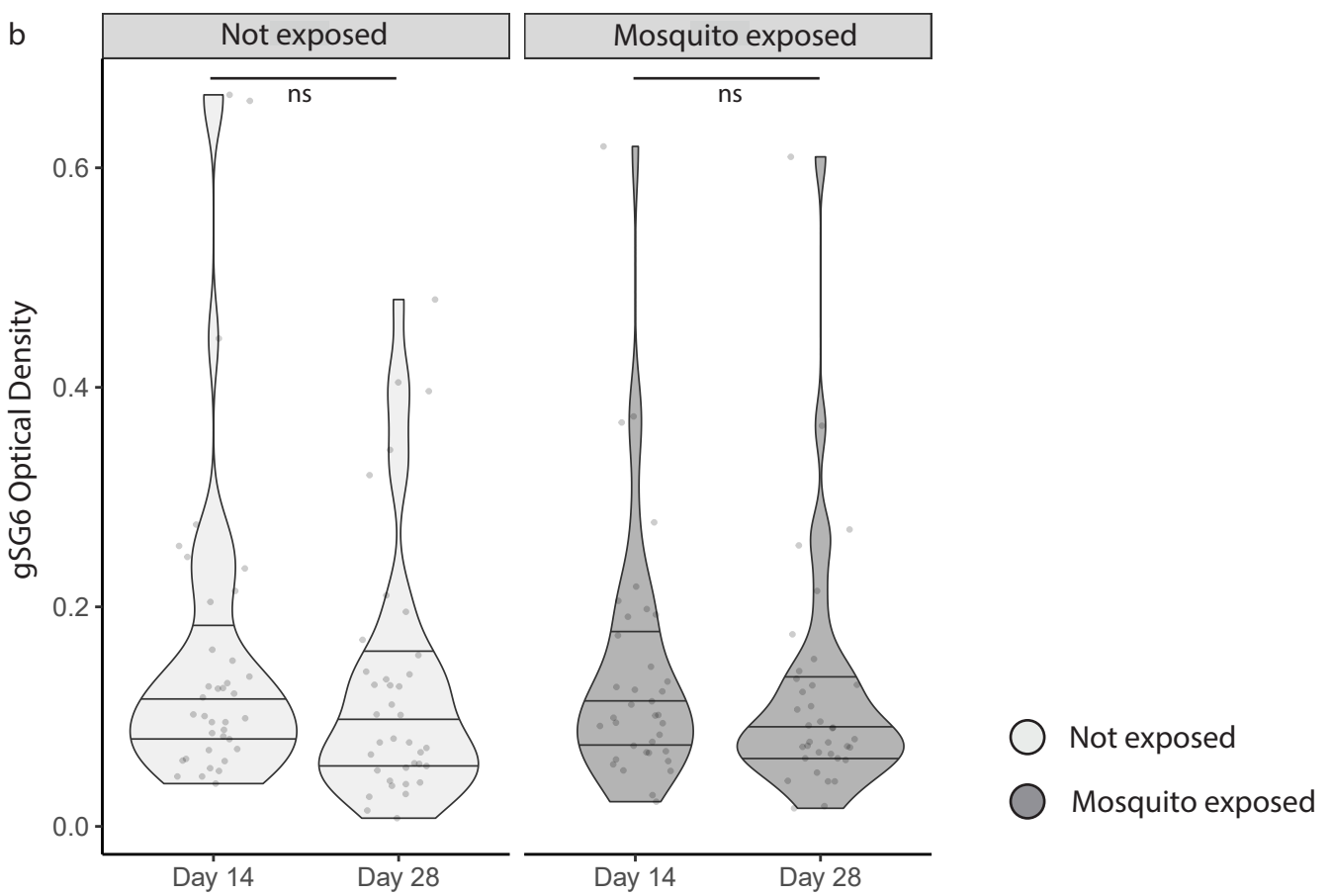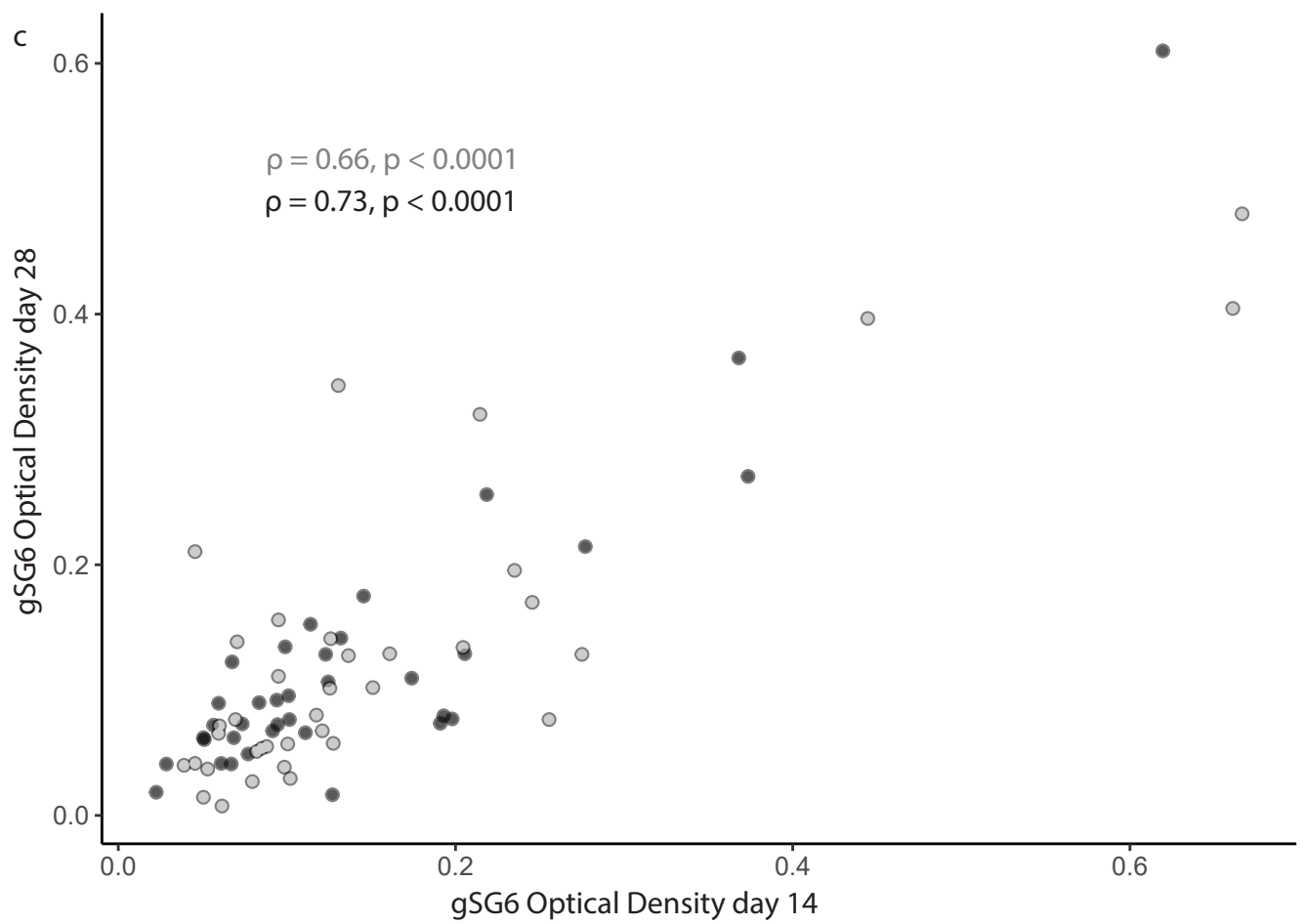

Supplement: Supplementary Figure S6 — Anti-gSG6 IgG responses upon exposure to mosquitoes in the longitudinal cohort study. At day 14 of the follow-up half of the Burkinabe cohort (n = 59) was exposed to 60 uninfected mosquito bites. Antibodies against gSG6 were measured at baseline (day 14) and two weeks post-exposure (day 28) using ELISA and expressed as 450nm OD-values. a) Anti-gSG6 antibodies per age category on day 14 and day 28 combined. Due to plasma availability, pair-wise comparisons of 73 individuals could be made; 18 individuals aged 5-9 years old, 36 individuals aged 10 to 15 years old, and 19 individuals aged 16 years or above. b) Anti-gSG6 responses from exposed (n=36 individuals) versus control individuals (n=37 individuals) on 14 and day 28. c) The relation between an individual’s anti-gSG6 antibodies at baseline (day 14) and 2 week later (day 28) for exposed versus control individuals. Spearman rho indicates the correlation between day 14 and day 28 anti-gSG6 antibodies for the two cohort types separately. [file mmc6.pdf]
