# Supplementary material for: Plasmodium falciparum gametocyte production correlates with genetic markers of parasite replication but is not influenced by experimental exposure to mosquito biting
Source: eBioMedicine. 2024 Jun 19;105:105190. doi: 10.1016/j.ebiom.2024.105190 (PMC11239461; doi:10.1016/j.ebiom.2024.105190)
Supplement: Supplementary Tables [file mmc7.pdf]

Supplementary Tables

Supplementary Table 1: Primer sequences for detecting ring-stage parasites (sbp-1), female (CCp4) and male (PfMGET) gametocytes.

**Target**	**GeneID**	**Primer (5'-3')**	**Intron spanning**	**Primer concentration**
sbp-1 forward	PF3D7_0501300	GCAAAACAAGCCGTACATGTTG	Yes	225 nM
sbp-1 reverse		TTGCTAGGTAATATCCTTTTCTTTTTCC		
uce forward	PF3D7_0812600	GGTGTTAGTGGCTCACCAATAGG	No	250 nM
uce reverse		GTACCACCTTCCCATGGAGTA		
CCp4 forward	PF3D7_0903800	CACATGAATATGAGAATAAAATTG	Yes	900 nM
CCp4 reverse		TAGGCGAACATGTGGAAAG		
CCp4 probe		TexasRed-AGCAACAACGGTATGTGCCTTAAAACG-BHQ2		125 nM
PfMGET forward	PF3D7_1469900	CGGTCCAAATATAAAATCCTG	Yes	225 nM
PfMGET reverse		TGTGTAACGTATGATTCATTTTC		
PfMGET probe		FAM-CAGCTCCAGCATTAAAACAC-BHQ1		125 nM

Supplementary Table 2: Primer sequences of 11 potential gametocyte commitment markers.

Target	GeneID	Primer	Intron spanning	Primer concentration
ap2-g_forward	PF3D7_1222600	TGGTGGTAATAAGAACAACAGAGGT	No	900 nM
ap2-g_reverse		CCATCATAATCTTCTTCTTCGTCG		
gdv1_forward	PF3D7_0935400	TAGGCGTCGAAATAGTGCTAGTAGAAA	Yes	900 nM
gdv1_reverse		GTCCTCACAACCAGCATCATTAGTA		
gexp-5_forward	PF3D7_0936600	GGGCTGTTATGTATATTTTTATTGAATTTT	Yes	900 nM
gexp-5_reverse		CATTCGTTTCATTTTCACCACTTC		
sir2a_forward	PF3D7_1328800	GGGAATGTATTTGAAGCAGT	No	900 nM
sir2a_reverse		CGATGTGCCAATTACTAAAA		
surfin1.2_forward	PF3D7_0113600	TTTTTCCCTCGATCTCCGCG	No	900 nM
surfin1.2_reverse		GGGTTTGGCCGTACTCTACT		
surfin13.1_forward	PF3D7_1301800	ACCCGAAGTACAACATCTCC	No	900 nM
surfin13.1_reverse		TCTCCACGAGTTCCAAGTTTT		
pfpeg4_forward	PF3D7_1016900	GCTGCTGTTGCTTTGGCTAT	No	900 nM
pfpeg4_reverse		TCCTCAGAGTCGGATCCATCA		
pfg27_forward	PF3D7_1302100	GCGTATCATGAACGACAAGAA	No	900 nM
pfg27_reverse		ACGGGTAAAGCAGGTATTGG		
msrp1_forward	PF3D7_1335000	TCACAAACGTCTAGTCCCGA	No	900 nM
msrp1_reverse		TGGTCTTGGTGATTCTGGCA		
gexp-2_forward	PF3D7_1102500	CTGATAATTCTTCATAAACGGGTG	No	900 nM
gexp-2_reverse		ATCTTAAAGAGTGTAAAACAGCAGA		
asRNA *gdv1_forward*	PF3D7_0935390	CGTCAGCATCATCATCTTC	No	900 nM
asRNA *gdv1_reverse*		ACTATTAGATATTATAATGGAAAC		

Supplementary Table 3: Genetic markers and their previously described function and parasite phase of expression.

**Target**	**GeneID**	**Function**	**Phase**	**References**
ap2-g	PF3D7_1222600	Transcription factor required for gametocyte commitment	Asexual blood stage	[6, 25]
gdv1	PF3D7_0935400	Antagonizes HP1-dependent gene silencing of *Ap2g*	Asexual blood stage and early gametocytes	[26, 27, 28]
asRNA g*dv1*	PF3D7_0935390	Inhibits *Gdv1* transcription	Asexual blood stage	[26, 54]
gexp-5	PF3D7_0936600	Exported protein independent of a*p2g*	Early and mature stage gametocytes	[24, 30, 31]
gexp-2	PF3D7_1102500	Interacts with erythrocyte cytoskeleton during sexual development	Asexual blood stage	[32, 33, 34, 35]
surfin1.2	PF3D7_0113600	Pseudogene, unknown function	Asexual blood stage	[24]
surfin13.1	PF3D7_1301800	Pseudogene, unknown function	Asexual blood stage	[24]
msrp1	PF3D7_1335000	Unknown function	Asexual blood stage and early gametocytes	[4, 24, 36]
pfpeg4	PF3D7_1016900	Encodes protein ETRAMP10.3, located on parasitophorous vacuole membrane	Early stage gametocytes (stage I and II)	[31, 37, 38]
pfg27	PF3D7_1302100	Encodes gamete antigen 27/25, contributes to cell integrity	Early stage gametocytes (stage I)	[31, 38, 39, 40]
sir2a	PF3D7_1328800	Inhibits DNA synthesis and multiplication rate	Asexual blood stage	[41, 42]

Supplementary Table 4: Marker transcripts *in vitro*. Transcripts of 11 genetic markers were detected using qPCR in percoll gradient synchronized NF54 3D7 *P. falciparum* culture material. Blood-stage parasites were harvested at 10, 20, 30, and 40 hours to respectively collect early and late-ring stage parasites, mature trophozoites, and schizonts. Material was treated with N-acetylglucosamine to eliminate asexual parasites and obtain mature gametocytes at day 16 post synchronization. Transcript levels were assessed using two concentrations per parasite stage. Per reaction, CT levels are indicated and an N/A is given when zero transcripts were detected.

	ap2-g	*pfpeg4*	*pfg27*	asRNA *gdv1*	*gexp-5*	*gexp-2*	*sir2a*	*surfin13.1*	*surfin1.2*	*msrp1*	*gdv1*
**Early ring-stage**											
10^6 / mL	30.92	33.81	32.07	N/A	32.44	N/A	35.28	30.43	34.00	30.3	25.94
10^5 / mL	32.61	N/A	N/A	33.12	28.92	N/A	37.08	N/A	N/A	N/A	29.89
**Late ring-stage**											
10^6 / mL	28.13	27.74	27.14	N/A	20.03	31.42	36.24	28.62	31.11	29.23	25.98
10^5 / mL	34.24	31.47	31.06	N/A	23.61	32.77	35.59	32.53	N/A	N/A	N/A
**Trophozoites**											
10^6 / mL	33.4	31.11	30	N/A	28.83	33.05	36.5	N/A	N/A	N/A	29.14
10^5 / mL	N/A	N/A	31.75	N/A	31.78	N/A	N/A	N/A	N/A	N/A	33.41
**Schizonts**											
10^6 / mL	33.5	29.79	25.99	33.23	26.87	31.94	34.96	N/A	N/A	36.04	25.11
10^5 / mL	N/A	32.12	30.54	N/A	30.12	N/A	38.54	N/A	N/A	N/A	29.49
**StV gametocytes**											
10^6 / mL	30.07	24.29	21.83	26.74	21.5	31.56	35.28	33.49	32.02	30.72	22.74
10^5 / mL	31.17	28.35	25.88	30.37	23.71	N/A	40.91	N/A	32.94	33.34	N/A

Supplementary Table 5: Genetic marker levels in a pilot subset of samples from Burkina Faso. To assess marker transcript suitability in field samples, we selected a subset (n=7) of samples from the longitudinal follow-up study in Burkina Faso. This pilot selection included samples negative for both ring-stage parasites (sbp-1) and gametocytes (n=3, sample IDs 1-3), samples that were ring-stage negative yet gametocyte positive (n=2, background signal in StV gametocytes, samples IDs 4 and 5), and samples without gametocytes yet with ring-stage parasites and of which the following sample was gametocyte positive (n=2, 'clean commitment', sample IDs 6 and 7). Values indicate CT-values per reaction, N/A indicates no CT was detected. Reactions with wrong melt-temperatures were excluded.

	**SampleID**						
	**1**	**2**	**3**	**4**	**5**	**6**	**7**
Gametocytes on T	Negative	Negative	Negative	Positive	Positive	Negative	Negative
Gametocytes on T+14	Negative	Negative	Negative	Positive	Positive	Positive	Positive
Sbp-1 on T	Negative	Negative	Negative	Negative	Negative	Positive	Positive
ap2-g	N/A	N/A	N/A	N/A	N/A	26.02	33.17
msrp-1	38.38	N/A	N/A	N/A	N/A	27.96	32.75
pfpeg4	36.9	N/A	N/A	32.26	N/A	31.35	30.21
pfg27	31.88	N/A	N/A	30.69	32.81	24.84	24.19
asRNA gdv1	31.74	N/A	N/A	no melt	N/A	30.13	32.02
gexp-5	no melt	N/A	N/A	30.79	30.93	22.5	27.38
gexp-2	N/A	N/A	N/A	31.52	N/A	N/A	31.7
sir2a	N/A	N/A	N/A	N/A	N/A	29.07	30.28
surfin1.2	N/A	N/A	N/A	N/A	N/A	26.01	31.44
surfin13.1	N/A	N/A	N/A	N/A	N/A	23.78	32.63
gdv1	no melt	N/A	N/A	33.57	34.13	24.53	26.26

Supplementary Table 6: Demographics of study participants from controlled human malaria infection (CHMI) study in the Netherlands. Study participants were all aged between 20 and 30 years. Age is reported as the median of total study participants or by sex assigned at birth.

	Number of participants (N)	Age in years (median, IQR)
**Total participants**	24	24 (20 - 26)
Male	15	24 (20.5 – 26)
Female	9	21 (20 – 25)

Supplementary Table 7: Demographics of asymptomatic study participants from the longitudinal cohort study in Burkina Faso. Age is reported in age categories and participant sex categories indicate the sex assigned at birth. Individuals aged 5 years or above were eligible for screening.

	Number of participants (N)	Male (N)	Female (N)
**Total participants**	120	65	55
5-9 years old	30	16	14
10-15 years old	59	32	27
16 years or above	31	17	14

Supplementary Table 8: Ring-stage parasite and gametocyte prevalence at enrolment in the longitudinal cohort study in Burkina Faso, separated by age category.

	Total	Positives	
	N	n	%
**Ring-stage parasites**			
Enrolment observations	115	112	97.4
5-9 years	29	28	96.6
10-15 years	57	56	98.2
16 years and above	29	28	96.6
**Mature gametocytes**			
Enrolment observations	115	107	93.0
5-9 years	29	27	93.1
10-15 years	57	51	89.5
16 years and above	29	29	100.0

Supplementary Table 9: Future ring-stage parasites in relation to antibody responses against antigens that reflect cumulative and short-term exposure to *Plasmodium falciparum* parasites. Antibody responses against antigens associated with cumulative exposure (AMA-1, MSP-1.19, GLURP.R2) and with exposure in past 6 months (Rh2.2030, GEXP18, Etramp5.Ag1) were determined at baseline in the Burkina Faso cohort; at day 0 (n = 115) or day 14 (n = 4) of follow-up. A multilevel model was used with an interaction term between antibody response for a specific antigen and ring-stage parasite density on timepoint T to assess the relation to ring-stage parasite density 14 days later (i.e. timepoint T^{+14}). Ring-stage parasite density at T^{+14} was the model outcome variable. Antibody responses against the six antigens were included as categorical variables in separate models. For each antigen separately, the first quartile (lowest 25% in mean fluorescent intensity) was used as reference category and compared to the fourth quartile (upper 25%). For every antigen, the estimate (β) indicates the difference in the relation between current and future ring-stage parasites (on timepoint T^{+14}) for the upper 25% compared to the lower 25% with associated P-values. All models accounted for an effect of age (in categories 5-9 years, 10-15 years, 16 years and above).

	Ring-stage parasites on timepoint T^{+14}	
Ring-stage parasites on timepoint T	**Estimate**	**p-value**
**AMA-1**		
Quartile 1 (reference)	0.44	
Quartile 4	-0.29	0.454
**MSP-1.19**		
Quartile 1 (reference)	0.12	
Quartile 4	+0.48	0.070
**GLURP.R2**		
Quartile 1 (reference)	0.001	
Quartile 4	+0.66	0.107
**Rh2.2030**		
Quartile 1 (reference)	0.47	
Quartile 4	-0.35	0.429
**GEXP18**		
Quartile 1 (reference)	0.37	
Quartile 4	-0.05	0.874
**Etramp5.Ag1**		
Quartile 1 (reference)	0.42	
Quartile 4	-0.39	0.21

Supplementary Table 10: Future gametocyte densities in relation to combinations of marker transcript densities. To assess whether combinations of marker transcripts at timepoint T relate to gametocyte densities 14 days later (T^{+14}), a multilevel model was used with combinations of three markers (*ap2-g, gexp-2, sir2a)* at T as a predictor variable and gametocyte density at T^{+14} as an outcome variable. The model accounted for concurrent ring-stage parasites (*sbp-1*) and gametocytes, and included a random person effect to account for multiple observations from the same individual. Fold change indicates the change in outcome variable when the predictor variable would increase 10 fold, 95% confidence interval (CI) indicates the range of fold changes. p-values below 0.05 were considered significant and R^2 indicates the goodness of fit.

	Mature gametocyte density per µL on T^{+14}			
Transcripts per µL on T	**Fold change**	**95% CI**	**p-value**	**R^2**
gexp-2 + sir2a				
gexp-2	1.26	0.98 – 1.62	0.069	0.485
sir2a	1.47	1.06 – 2.04	0.022	
sir2a + ap2-g				
sir2a	1.59	1.17 – 2.17	0.004	0.490
ap2-g	0.09	1.14 – 1.38	0.151	
ap2-g + gexp-2				
ap2-g	1.12	0.92 – 1.35	0.253	0.474
gexp-2	1.34	1.06 – 1.71	0.016	
sir2a + surfin1.2				
sir2a	1.61	1.19 – 2.19	0.003	0.490
surfin1.2	1.17	0.98 – 1.41	0.087	
sir2a + surfin13.1				
sir2a	1.62	1.19 – 2.20	0.002	0.489
surfin13.1	1.18	0.97 – 1.44	0.101	
ap2-g + surfin1.2				
ap2-g	1.27	1.01 – 1.60	0.045	0.755
surfin1.2	1.13	0.91 – 1.41	0.277	
ap2-g + surfin13.1				
ap2-g	1.35	1.09 – 1.66	0.007	0.754
surfin13.1	1.03	0.83 – 1.29	0.761	
gexp-2 + surfin1.2				
gexp-2	1.38	1.09 – 1.75	0.008	0.475
surfin1.2	1.13	0.94 – 1.37	0.195	
gexp-2 + surfin13.1				
gexp-2	1.38	1.09 – 1.75	0.008	0.473
surfin13.1	1.12	0.91 – 1.38	0.275	
surfin1.2 + surfin13.1				
surfin1.2	1.16	0.89 – 1.52	0.276	0.446
surfin13.1	1.13	0.84 – 1.51	0.416	

Supplementary Table 11: Present marker transcript density in relation to future gametocyte density, whilst accounting for present *uce* transcripts. To assess whether marker transcripts at timepoint T relate to gametocyte densities 14 days later (T^{+14}), a generalized additive model was used. Gametocyte density at timepoint T^{+14} was used as the model outcome variable. Marker (*ap2-g, sir2a, surfin1.2, gexp2, surfin13.1)* transcripts were used as a predictor variable in separate models. The model accounted for concurrent *uce* transcripts separately. The model also accounted for an effect of time, age (in categories), and included a random person effect to account for multiple observations from the same individual. Fold changes represent the fold change (= 10^{β}) in outcome variable when the indicated predictor variable increased by 10-fold, the 95% confidence interval (CI) indicates the range of fold-changes, p-values below 0.05 were considered significant and R^2 indicates the goodness of fit.

	Gametocyte density per µL on T^{+14}			
Marker transcripts per µL on T	**Fold change**	**95% CI**	**p-value**	**R^2**
ap2-g	1.35	1.14 - 1.60	0.003	0.766
sir2a	1.48	1.15 - 1.89	0.006	0.488
gexp-2	1.37	1.10 - 1.71	0.010	0.476
surfin1.2	1.23	1.03 - 1.47	0.032	0.451
surfin13.1	1.23	1.00 - 1.51	0.047	0.448

Supplementary Table 12: Gametocyte formation in relation to mosquito exposure. A multilevel model with interaction term between exposure and ring-stage density was used, whilst accounting for gametocyte densities on day 14 and day 20, since these could persist and contribute to the pool of day 28 gametocytes. An additional effect of age category was also accounted for. Estimates indicate the direction and strength of the association for the reference category (not exposed control cohort) and the relative difference in estimate for the mosquito exposed cohort. P-value indicates whether this difference (the interaction term) was significant.

Ring-stage parasite density per µL on day 14	**Estimate**	**p-value**
Control (reference)	0.22	
Mosquitoes exposed	-0.11	0.379

Supplementary Table 13: Relation between mosquito exposure and consequent genetic marker transcript levels. On day 14 of the follow-up period, 49.2% (n=59) of the Burkinabe cohort were exposed to uninfected *Anopheles gambiae* bites through direct skin-feeding. A multilevel model was used with an interaction term between mosquito exposure and transcript density on day 14 to assess its relation to marker density on day 20. Individuals that were not exposed to mosquito bites were used as reference. The model also accounted for concurrent gametocyte and ring-stage parasite densities on day 14. An additional effect of age category was also accounted for. Estimates (β) for the reference category indicate the association between marker density on day 14 and marker density on day 28. Estimates for the exposed category indicate the difference in estimate for the exposed individuals compared to the reference category. P-values indicate whether this difference was significant.

	Transcripts per µL on day 20	
Transcripts per µL on day 14	**Estimate**	**p-value**
ap2-g		
Control (reference)	0.01	
Mosquitoes exposed	+0.18	0.294
sir2a		
Control (reference)	0.34	
Mosquitoes exposed	-0.15	0.469
gexp-2		
Control (reference)	0.49	
Mosquitoes exposed	-0.50	0.052
surfin1.2		
Control (reference)	0.05	
Mosquitoes exposed	+0.12	0.461
surfin13.1		
Control (reference)	0.11	
Mosquitoes exposed	+0.03	0.866

Supplementary Table 14: Outcomes of feeding assays performed on day 14 and day 28 of the longitudinal follow-up study in Burkina Faso. On day 14, 59 individuals participated in membrane-feeding and skin-feeding, the other individuals participated in membrane-feeding only. On day 28, membrane feeding assays were performed on the entire cohort.

	**Infectious feeds**		**Infected mosquitoes**	
	Mosquito exposed	Control	Mosquito exposed	Control
**Day 14 (total)**	11		70	
Skin-fed	4/11 (36.4%)	-	17/70 (24.3%)	-
Membrane feeding	3/11(27.3%)	4/11(36.4%)	18/70 (25.7%)	35/70 (50.0%)
**Day 28 (total)**	5		30	
Membrane feeding	2/5 (40.0%)	3/5 (60.0%)	16/30 (53.3%)	14/30 (46.7%)
