# Supplementary material for: Plasmodium falciparum gametocyte production correlates with genetic markers of parasite replication but is not influenced by experimental exposure to mosquito biting
Source: eBioMedicine. 2024 Jun 19;105:105190. doi: 10.1016/j.ebiom.2024.105190 (PMC11239461; doi:10.1016/j.ebiom.2024.105190)
Supplement: Appendix [file mmc9.docx]

**Appendix**

**Statistical methods**

The association between genetic marker transcript levels at timepoint T and total ring-stage density 14 days later (T^+14^) was determined by general additive mixed models for the five markers separately or in combination, whilst accounting for the influence of time and concurrent ring-stage and gametocyte densities and participant age; correlations between observations from the same individual were accounted for by a participant-specific random effect. A similar model was used to determine the association between marker transcript levels at timepoint T and gametocyte densities 14 days later (T^+14^). Outcome variables of the models (parasite or gametocyte densities at T^+14^) were all continuous, and predictor variables were either continuous (marker densities, concurrent gametocyte, ring-stage or *uce* transcript densities) or categorical (age category, time). Marker densities were considered the predictor variables of interest. All generalized additive models were used without any smoothing splines, resulting in a model identical to a linear mixed effects model that assumes normality and constant variance of the residuals. To ensure normality of the residuals from the models, a log10 transformation of continuous density variables (ring-stage parasites, gametocytes, marker transcripts) was used and the distribution of the residuals are visualized below (Appendix Figure 1 and 2). Additionally it is shown that the log10 transformation results in the continuous densities themselves presenting a more normal distribution (Appendix Figure 3).

The estimate (β) of the association between log10 transformed predictor and log10 transformed outcome variable was used to calculate the fold change (10^β^). Fold change can be interpreted as follows: when a predictor variable increases 10-fold, the outcome variable changes by a fold change of 10^β^. In models that shared an outcome variable, we accounted for multiple testing using the Benjamini Hochberg approach. The 95% confidence intervals (CI) indicate the range in fold-changes, and the p-values indicate significance of the association. In models that shared an outcome variable, we accounted for multiple testing using the Benjamini Hochberg approach which allows for a maximum 5% type 1 error rate amongst the set of rejected hypotheses. In models with only accepted null hypotheses (P > 0.05), we did not adjust for multiple testing since this would not change significance levels below the 5% type 1 error rate.


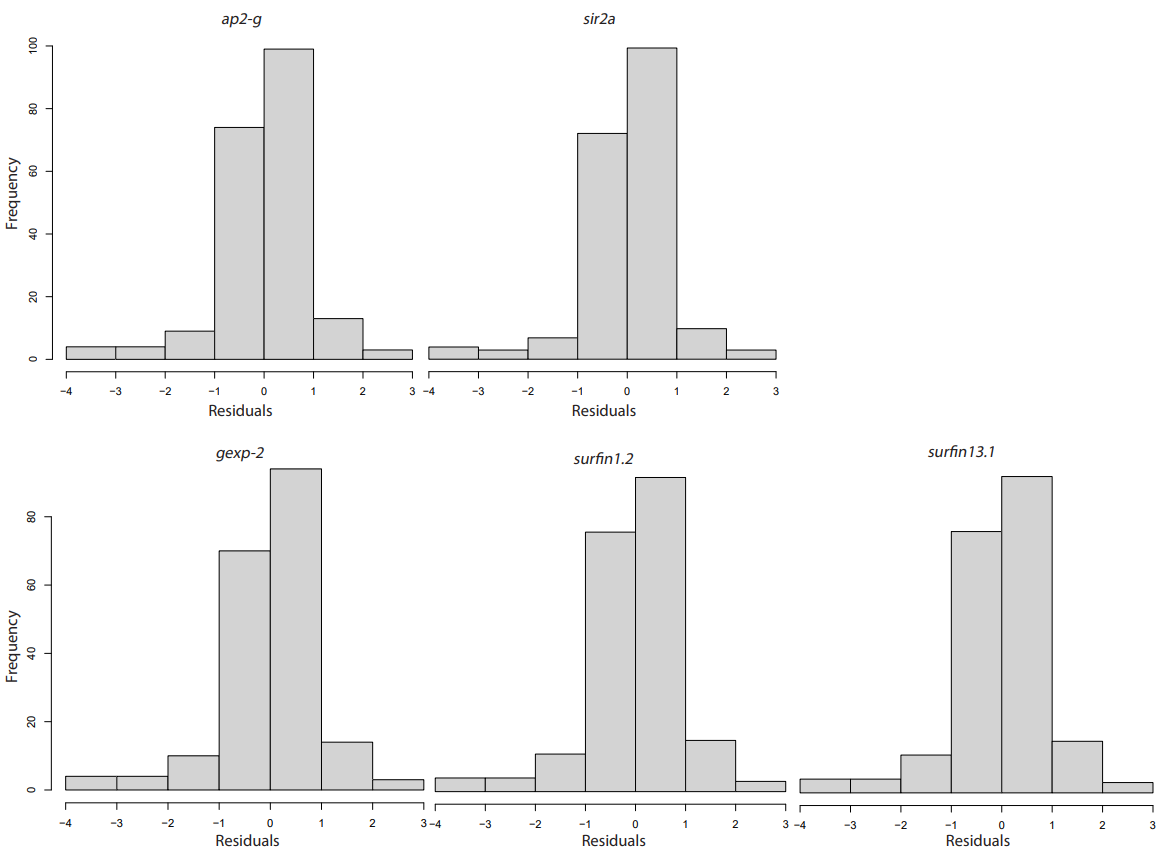


**Appendix Figure 1.** Histograms of the residuals from the model predicting ring-stage parasites (*sbp-1*) at timepoint T^+14^. Headings indicate the marker included in the model. For every marker separately, a generalized additive model was used with ring-stage parasites at timepoint T^+14^ as outcome variable, and marker density, ring-stage parasite density and gametocyte density at timepoint T as predictor variables. The model also accounted for an effect of age (in categories) and time, and included a random person-effect.


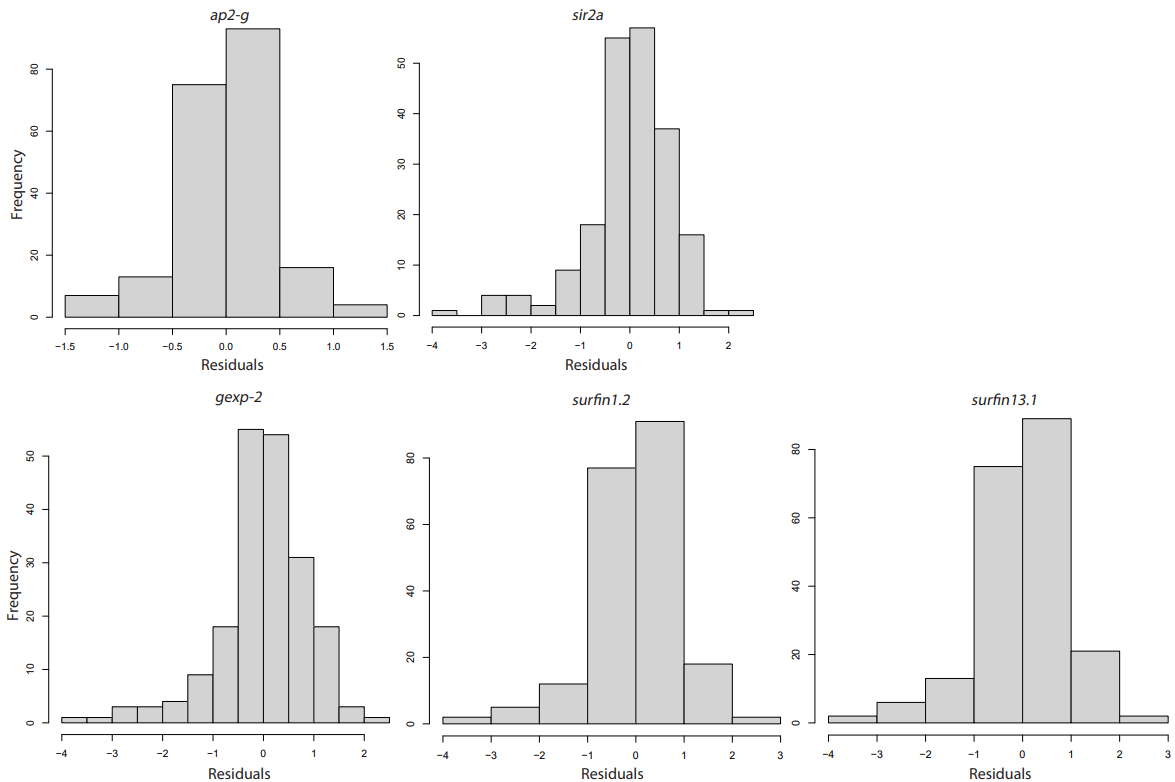


**Appendix Figure 2.** Histograms of the residuals from the model predicting gametocyte density at timepoint T^+14^. Headings indicate the marker included in the model. For every marker separately, a generalized additive model was used with gametocytes at timepoint T^+14^ as outcome variable, and marker density, ring-stage parasite density and gametocyte density at timepoint T as predictor variables. The model also accounted for an effect of age (in categories) and time, and included a random person-effect.


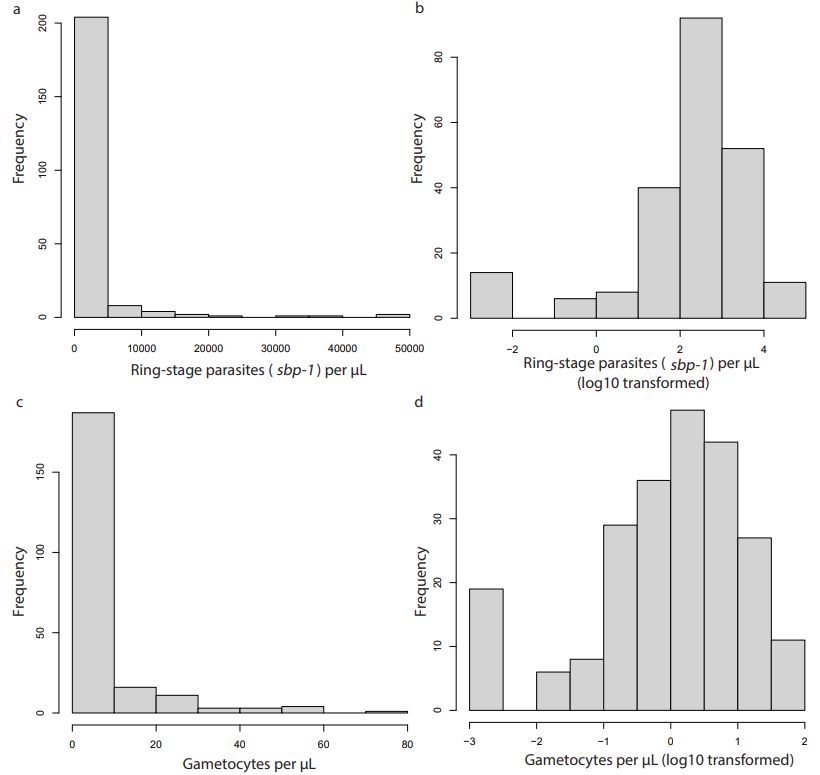


**Appendix Figure 3.** Histograms of ring-stage parasites (*sbp-1*) and mature gametocytes, the main model outcome variables. a) Histogram of untransformed ring-stage parasites at timepoint T^+14^ b) Histogram of log10 transformed ring-stage parasites at timepoint T^+14^ . c) Histogram of mature gametocyte density at timepoint T^+14^ without any data transformation. d) Histogram of log10 transformed gametocyte density at timepoint T^+14^
